# Functional EF-Hands in Neuronal Calcium Sensor GCAP2 Determine Its Phosphorylation State and Subcellular Distribution *In Vivo*, and Are Essential for Photoreceptor Cell Integrity

**DOI:** 10.1371/journal.pgen.1004480

**Published:** 2014-07-24

**Authors:** Natalia López-del Hoyo, Santiago López-Begines, Jose Luis Rosa, Jeannie Chen, Ana Méndez

**Affiliations:** 1 Bellvitge Biomedical Research Institute (IDIBELL), Barcelona, Spain; 2 Department of Physiological Sciences II, University of Barcelona-Bellvitge Health Science Campus, Barcelona, Spain; 3 Department of Cell and Neurobiology, Zilkha Neurogenetic Institute, Keck School of Medicine, University of Southern California, Los Angeles, California, United States of America; 4 Department of Pathology and Experimental Therapeutics, University of Barcelona-Bellvitge Health Science Campus, Barcelona, Spain; University of California San Diego, United States of America

## Abstract

The neuronal calcium sensor proteins GCAPs (guanylate cyclase activating proteins) switch between Ca^2+^-free and Ca^2+^-bound conformational states and confer calcium sensitivity to guanylate cyclase at retinal photoreceptor cells. They play a fundamental role in light adaptation by coupling the rate of cGMP synthesis to the intracellular concentration of calcium. Mutations in GCAPs lead to blindness. The importance of functional EF-hands in GCAP1 for photoreceptor cell integrity has been well established. Mutations in GCAP1 that diminish its Ca^2+^ binding affinity lead to cell damage by causing unabated cGMP synthesis and accumulation of toxic levels of free cGMP and Ca^2+^. We here investigate the relevance of GCAP2 functional EF-hands for photoreceptor cell integrity. By characterizing transgenic mice expressing a mutant form of GCAP2 with all EF-hands inactivated (EF^−^GCAP2), we show that GCAP2 locked in its Ca^2+^-free conformation leads to a rapid retinal degeneration that is not due to unabated cGMP synthesis. We unveil that when locked in its Ca^2+^-free conformation *in vivo*, GCAP2 is phosphorylated at Ser201 and results in phospho-dependent binding to the chaperone 14-3-3 and retention at the inner segment and proximal cell compartments. Accumulation of phosphorylated EF^−^GCAP2 at the inner segment results in severe toxicity. We show that in wildtype mice under physiological conditions, 50% of GCAP2 is phosphorylated correlating with the 50% of the protein being retained at the inner segment. Raising mice under constant light exposure, however, drastically increases the retention of GCAP2 in its Ca^2+^-free form at the inner segment. This study identifies a new mechanism governing GCAP2 subcellular distribution *in vivo*, closely related to disease. It also identifies a pathway by which a sustained reduction in intracellular free Ca^2+^ could result in photoreceptor damage, relevant for light damage and for those genetic disorders resulting in “equivalent-light” scenarios.

## Author Summary

Visual perception is initiated at retinal photoreceptor cells, where light activates an enzymatic cascade that reduces free cGMP. As cGMP drops, cGMP-channels close and reduce the inward current –including Ca^2+^ influx– so that photoreceptors hyperpolarize and emit a signal. As the light extinguishes, cGMP levels are restored to reestablish sensitivity. cGMP synthesis relies on guanylate cyclase/guanylate cyclase activating protein (RetGC/GCAP) complexes. GCAPs link the rate of cGMP synthesis to intracellular Ca^2+^ levels, by switching between a Ca^2+^-free state that activates cGMP synthesis during light exposure, and a Ca^2+^-bound state that arrests cGMP synthesis in the dark. It is established that GCAP1 mutations linked to adCORD disrupt this tight Ca^2+^ control of the cGMP levels. We here show that a GCAP2 functional transition from the Ca^2+^-free to the Ca^2+^-loaded form is essential for photoreceptor cell integrity, by a non-related mechanism. We show that GCAP2 locked in its Ca^2+^-free form is retained by phosphorylation and 14-3-3 binding to the proximal rod compartments, causing severe cell damage. This study identifies a pathway by which a sustained reduction in intracellular free Ca^2+^ could result in photoreceptor damage, relevant for light damage and for those genetic disorders resulting in “equivalent-light” scenarios.

## Introduction

Guanylate-cyclase activating proteins (GCAPs) belong to the neuronal calcium sensor (NCS) family of proteins that display limited similarity to calmodulin. They confer Ca^2+^-sensitivity to guanylate-cyclase (Ret-GC) activity in retinal photoreceptor cells. GCAP1 and GCAP2 constitute the major species in mammals [1]–[3]. At rod and cone outer segments GCAPs form permanent complexes with Ret-GCs allowing short response times of cyclase modulation to fluctuations in intracellular Ca^2+^ concentration. GCAPs inhibit cyclase activity in their Ca^2+^-loaded form at the high free [Ca^2+^]_i_ characteristic of the dark steady-state, and switch to their activator state as they replace Ca^2+^ by Mg^2+^ when light reduces Ca^2+^ influx upon closing the cGMP-channels [4]. Light exposure results in up to a 10-fold decline in the intracellular free [Ca^2+^], from ∼250 nM in darkness to 23 nM in saturating light in mouse rod outer segments [5]. This Ca^2+^ decrease is first sensed at GC/GCAP complexes comprising GCAP1 and successively at those comprising GCAP2 [Ca^2+^ EC_50_ for GCAP1 ∼130 nM; for GCAP2 ∼50 nM, [6]], in a sequential mode of action referred to as a Ca^2+^-relay model [7]–[9]. Altogether, the rate of cGMP synthesis upon light exposure is stimulated up to ∼12-fold over its basal levels, serving to restore the cGMP levels and to reopen the channels during the recovery of the light response and light adaptation [10], [11].

Despite the importance of GCAPs-mediated Ca^2+^-feedback on cGMP synthesis in the control of sensitivity, deletion of GCAP1 and GCAP2 in mice does not lead to significant effects on retinal morphology, indicating that GCAPs are not essential for the development or maintenance of retinal organization [11]. However, mutations in the GCAP1 and GCAP2 genes have been linked to inherited autosomal dominant retinopathies. Ten heterozygous mutations in the GUCA1A gene encoding GCAP1 have been linked to autosomal dominant cone dystrophy (adCD), cone rod dystrophy (adCRD) or macular degeneration (adMD) [12]–[20]. One mutation in the GUCA1B gene, G157R, has been associated to autosomal dominant retinal dystrophies ranging from retinitis pigmentosa to macular degeneration [21].

Most of GCAP1 mutations map at EF-hand domains and affect Ca^2+^ coordination directly, such as D100E and N104K at EF-3 or L151F and E155G at EF-4 [13]–[15], [20], or map at the incoming or outgoing α-helixes in EF-3 and EF-4, such as E89K, Y99C, T114I, I143NT and G159V, causing conformational distortions that make Ca^2+^ binding less favorable [15], [17], [18]. These mutations shift the Ca^2+^ IC_50_ of GC activation to higher free [Ca^2+^], so that *in vitro* the mutant proteins fail to switch to the inhibitory state and lead to persistent activation of RetGC in the whole physiological range of [Ca^2+^]_i_ [15], [20], [22]–[24]. *In vivo*, as demonstrated for the Y99C and E155G GCAP1 mutations, the unabated cGMP synthesis results in abnormally high levels of cGMP and Ca^2+^ in rods, and the ensuing retinal degeneration can be significantly prevented by conditions that promote constitutive stimulation of PDE6 such as constant light exposure [23], [25], [26].

There are aspects of GCAPs that remain less understood, such as their Ca^2+^-dependent structural changes or the mechanisms that determine their cellular distribution. GCAP1 and GCAP2 are both myristoylated at the NH_2_-terminus. While myristoylation of GCAP1 not only affects the affinity of GCAP1 for Ret-GC and Ret-GC maximal activation, but also increases the Ca^2+^ sensitivity of Ret-GC inhibition at EF-4 [27], myristoylation of GCAP2 affects its overall structural stability without affecting Ret-GC regulation [28]. Both GCAP1 and GCAP2 form homodimers upon Ca^2+^ dissociation, with the capacity to dimerize in GCAP2 correlating with the ability to activate Ret-GC [29]. However, while in GCAP2 dimerization is reversed by Ca^2+^ binding, GCAP1 dimerization is resistant to the presence of Ca^2+^, implying a difference in their Ca^2+^-dependent conformational changes [29]. Overall, the Ca^2+^-free form of GCAP2 shows a higher tendency to aggregate than GCAP1. In addition, Ca^2+^-dependent conformational changes in GCAP2 have been shown to correlate with a site-specific phosphorylation at Ser201, the significance of which is not yet clear as it does not affect Ret-GC regulation *in vitro* [30].

Regarding their cellular localization, GCAP1 is more abundant at cone than at rod outer segments [31]. GCAP2 localizes primarily to rods and at lower levels in cones. In rods, GCAP2 localization is not restricted to rod outer segments. It is present at rod inner segments at about the same level, and at lower levels in more proximal compartments of the cell [32], [33]. At the synaptic terminal GCAP2 has been shown to interact with Ribeye, the major structural component of synaptic ribbons, and to lead to significant alterations of synaptic ribbon dynamics when overexpressed *in vivo* [34], [35]. The mechanisms that determine GCAPs subcellular distribution are largely unknown, but it was proposed that GCAPs are transported by vesicular trafficking guided by Ret-GCs, in a process assisted by RD3 [36], [37].

To study whether genetic mutations or light conditions that would preclude Ca^2+^-binding to GCAP2 would compromise rod cell viability by an analogous mechanism by which GCAP1 EF-hand mutations do, we set to study the effect of expressing in rods a mutant form of GCAP2 impaired to bind Ca^2+^: GCAP2 with all functional EF hands inactivated (bEF^−^GCAP2) [38]. Whereas *in vitro* bEF^−^GCAP2 shows a similar biochemical behavior as Y99C-GCAP1 [24], [38], we show that *in vivo* it leads to a rapid retinal degeneration by a mechanism independent of cGMP metabolism. *In vivo*, the protein accumulates at the inner segment, in a form that is largely incompetent to activate the cyclase. It binds to 14-3-3 protein isoforms due to enhanced phosphorylation at Ser201. We show that the cause of the pathology in bEF^−^GCAP2 mice is not constitutive activation of the cyclase, but rather the accumulation of the phosphorylated protein at the proximal compartments of the cell, likely in a conformationally unstable form stabilized by 14-3-3 binding, that ultimately causes extensive damage to the cell. We propose that this mechanism will contribute to the pathology of those inherited retinal dystrophies caused by mutations in different genes that share as an initial consequence of the mutation the sustained reduction of the intracellular concentration of Ca^2+^, the so-called “light-equivalent damage” scenarios.

## Results

### Transgenic expression of bEF^−^GCAP2 in mouse rods leads to progressive retinal degeneration

**Figure 1:**
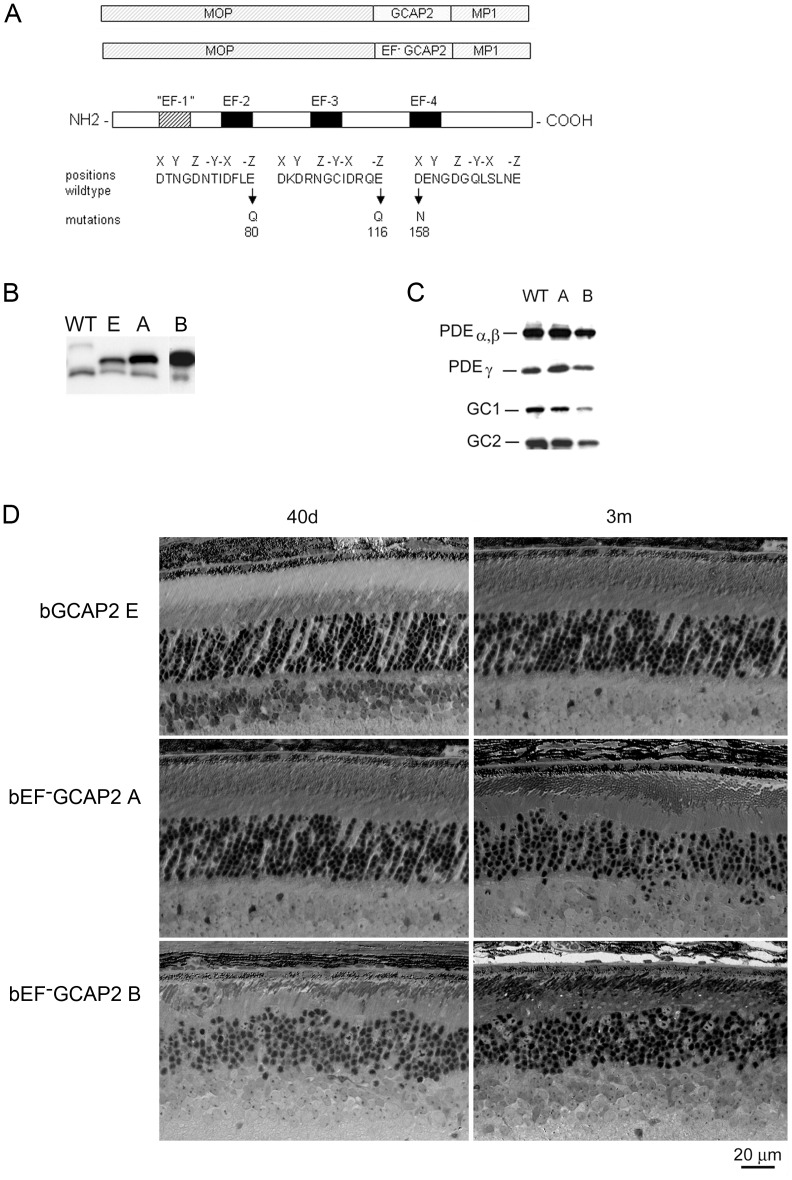
Transgene expression of bEF^−^GCAP2 in rods leads to retinal degeneration. **A**. Design of transgene expression vector. The cDNA of bovine GCAP2 with the three functional EF hands disrupted [GCAP2 (E80Q, E116Q, D158N) or bEF^−^GCAP2] was expressed under the mouse opsin promoter (MOP), with the polyadenilation signal of the mouse protamine 1 (MP1) gene. **B.** Western showing the level of expression of the transgene in bGCAP2 line E and bEF^−^GCAP2 lines A and B, compared to wildtype mice. Equivalent fractions of a retina were resolved by SDS-PAGE from wt (22 d of age), line E (40 d) and lines A (40 d) and B (22 d, showed from independent gel). An earlier time point was chosen for the strongest line (B) to reduce the effect that its rapid retinal degeneration has on total retinal protein content. Bovine and murine GCAP2 differ in size by three amino acids and can be distinguished by mobility. **C**. Compensatory changes in proteins involved in cGMP metabolism were not observed. The levels of PDEα,β and γ subunits, or GC1 and GC2 were mostly unaffected in mice from line A, whereas a reduction in all proteins was observed in line B at 22 d of age, due to the shortened outer segments in this line. **D.** Light micrographs of retinal sections from mice expressing bGCAP2 (line E) or bEF^−^GCAP2 transgene (lines A and B) in the GCAPs+/+ background at 40 d or 3 m.

To study the relevance of functional EF-hand domains in GCAP2 for protein activity and photoreceptor cell integrity *in vivo*, we expressed a mutant form of GCAP2 with inactivated EF hands: GCAP2(E80Q/E116Q/D158N), hereafter referred to as bEF^−^GCAP2, in the rod photoreceptors of transgenic mice (Fig. 1A). It was previously shown that inactivation of the three functional EF hands in bGCAP2 abolishes its capacity to bind Ca^2+^ [38]. To generate transgenic mice we expressed the cDNA of the bovine GCAP2 isoform, so that the transgene product could be distinguished from the endogenous murine form by SDS-PAGE electrophoretic mobility. To discriminate the effect of the mutations in GCAP2 from the effect that overexpression of GCAP2 might have on the cell, we included in the study a control transgenic line that expresses wildtype bovine GCAP2 (line E, Fig. 1A,B). This line was reported to express wildtype bovine GCAP2 at a ∼2∶1 ratio relative to endogenous GCAP2 [11].

We established two independent transgenic lines that expressed different levels of bEF^−^GCAP2. Line A expressed bEF^−^GCAP2 at a ratio of 2.76∶1 relative to endogenous GCAP2, whereas line B had a higher relative level of expression (3.85∶1 ratio), Fig. 1B and Fig. S1, see Methods.

To assess whether bEF^−^GCAP2 expression in rods causes compensatory changes in the expression levels of other proteins involved in cGMP metabolism, we compared the level of expression of PDE6 and Ret-GCs in retinal homogenates from wildtype and transgenic mice from lines A and B (Fig. 1C). Levels of PDEα, β and γ subunits, or GC1 and GC2 were mostly unaffected in mice from line A, whereas a reduction in all proteins was observed in line B at postnatal day 22 (p22), which can be explained by the dramatic shortening and disorganization of rod outer segments observed from a very early age in this line (Fig. 1D).

Mice expressing bEF^−^GCAP2 showed a progressive retinal degeneration whose severity correlated with the level of expression of the transgene. Figure 1D shows normal retinal morphology in the control transgenic line E at p40 and at 3 months of age. In contrast, clear signs of retinal degeneration were observed in mice expressing bEF^−^GCAP2 from lines A and B. Mice from line B, which express the highest levels of bEF^−^GCAP2, presented a substantial shortening of rod outer segments and a noticeable reduction of outer nuclear layer (ONL) thickness as early as p40, with ONL thickness reduced to 6–7 rows of nuclei. Mice from line A showed a slower progression of the disease, noticeable at 3 months, when the ONL thickness was reduced to 7–9 rows of nuclei.

Because expression of wildtype bGCAP2 did not cause retinal degeneration for up to one year of age in line E (results not shown), the retinal degeneration observed in mice from lines A and B likely results from distinctive properties of the mutant form of GCAP2 impaired to bind Ca^2+^. However, due to the different transgene expression levels, we could not exclude that the observed phenotype may result from overexpression of bGCAP2. To rule out this possibility, we bred the control line E to homozygosity, to obtain a line that expressed bGCAP2 to equivalent levels as mutant line A. This line showed normal outer segment length and organization, as well as normal outer nuclear layer thickness for up to six months of age when raised in cyclic light [34]. From these results we conclude that mutations that impair Ca^2+^ binding in GCAP2 lead to retinal degeneration *in vivo*.

### Retinal degeneration by bEF^−^GCAP2 is reproduced in the GCAPs −/− background, and correlates with the loss of visual function

In order to study the effects of the mutant protein on cell physiology, we bred the transgenic lines to GCAPs−/− mice, to obtain expression of bEF^−^GCAP2 or control bGCAP2 in the absence of the endogenous protein.

**Figure 2:**
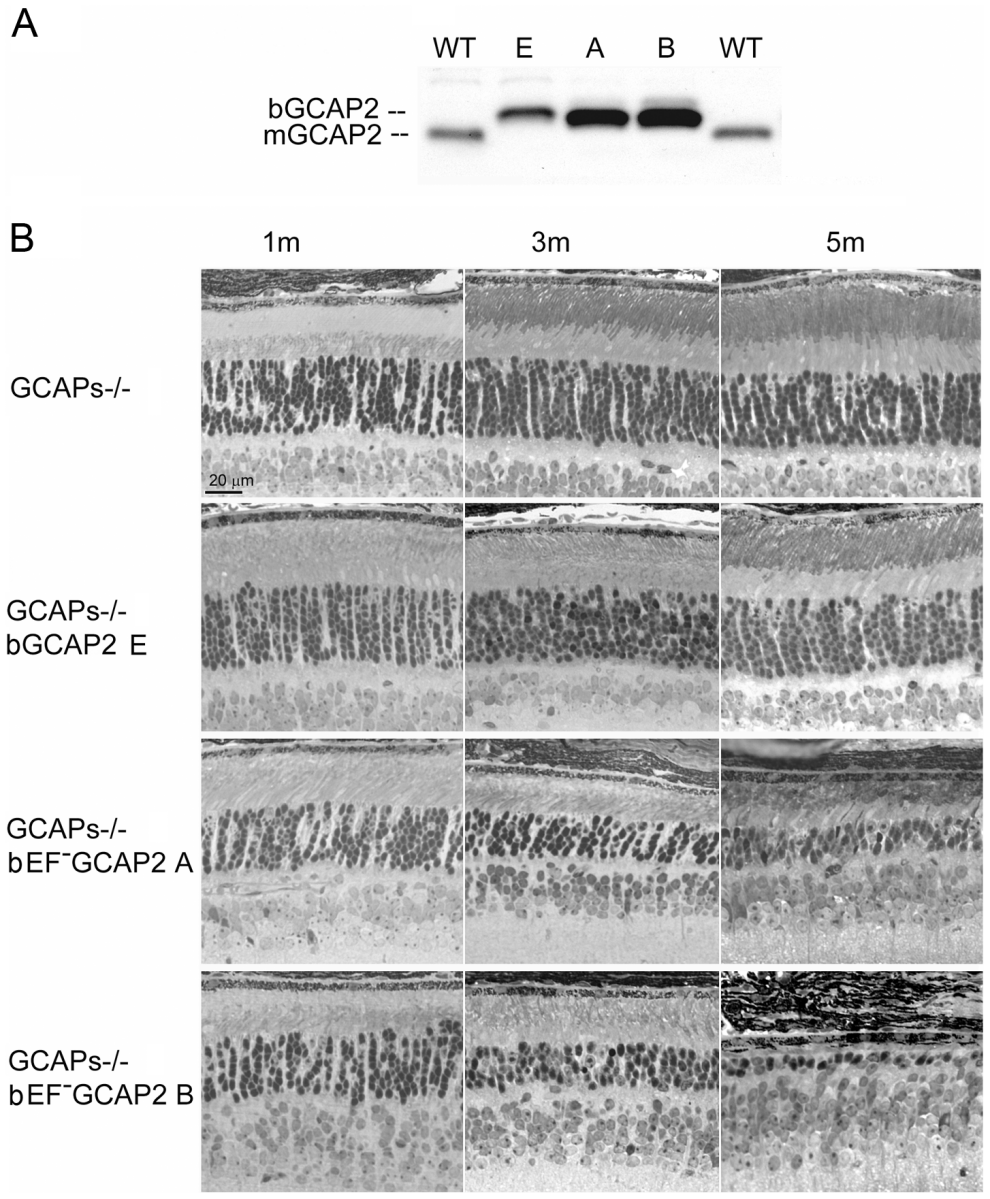
Transgenic expression and rate of retinal degeneration in the GCAPs−/− background. **A**. Levels of transgene expression in the GCAPs−/− background in mouse retinas from line E (ctrl bGCAP2) and lines A, B (bEF^−^GCAP2). Equal fractions of the retina were loaded from mice at 30 d of age. Transgene expression levels estimated in the GCAPs+/+ background were maintained in the GCAPs−/− background. **B**. Light micrographs of retinal sections from mice of the indicated genotypes at 1, 3 and 5 months of age, standard cyclic light rearing. Lines A and B show a progressive retinal degeneration in the GCAPs−/− background, that reduces the ONL thickness to 4–5 rows of nuclei at three months, and to 3 rows at five months (line A) or to a single row by five months of age (line B).

The relative levels of expression of the transgene in the independent transgenic lines were maintained in the GCAPs−/− background (Fig. 2A). Expression of bEF^−^GCAP2 in the GCAPs−/− background slightly accelerated the rate of retinal degeneration observed in the GCAPs+/+ background. Mice from the control lines GCAPs−/− and GCAPs−/− bGCAP2 E showed largely normal retinas with an outer nuclear layer (ONL) thickness of 10 rows of nuclei for up to 5 months of age (Fig. 2B), and preserved normal visual function when raised in cyclic light conditions as assessed by electroretinogram (ERG) [34]. In contrast, GCAPs−/− expressing bEF^−^GCAP2 showed a progressive retinal degeneration that correlated with loss of visual function (Fig. S2). In retinas from line B the ONL was reduced to six rows of nuclei and outer segments were much shorter than normal as early as p30 (Fig. 2B), when the A and B-wave amplitudes of ERG responses were half the size of normal responses from littermate controls (not shown). At 3 months of age the ONL was reduced to 4 rows of nuclei, and by 5 months it was limited to a single row. Mice were unresponsive to light (flat ERG traces) by 7 months (Fig. S2). A slightly slower retinal degeneration was observed in mice from line A that went from a normal outer nuclear layer thickness of 12 rows of nuclei at p30 to about 5 rows by 3 months of age. ERG responses of these mice resembled normal responses at very early ages, but A- and B-wave amplitudes were reduced by half by 4 months, correlating with a dramatic cell loss in these mice between p20 and 5 months of age (Fig. 2B and Fig. S2). Most of these mice are non responsive to light by ERG by 7–8 months (Fig. S2).

### bEF^−^GCAP2 protein accumulates in inactive form at the inner segment of the cell

**Figure 3:**
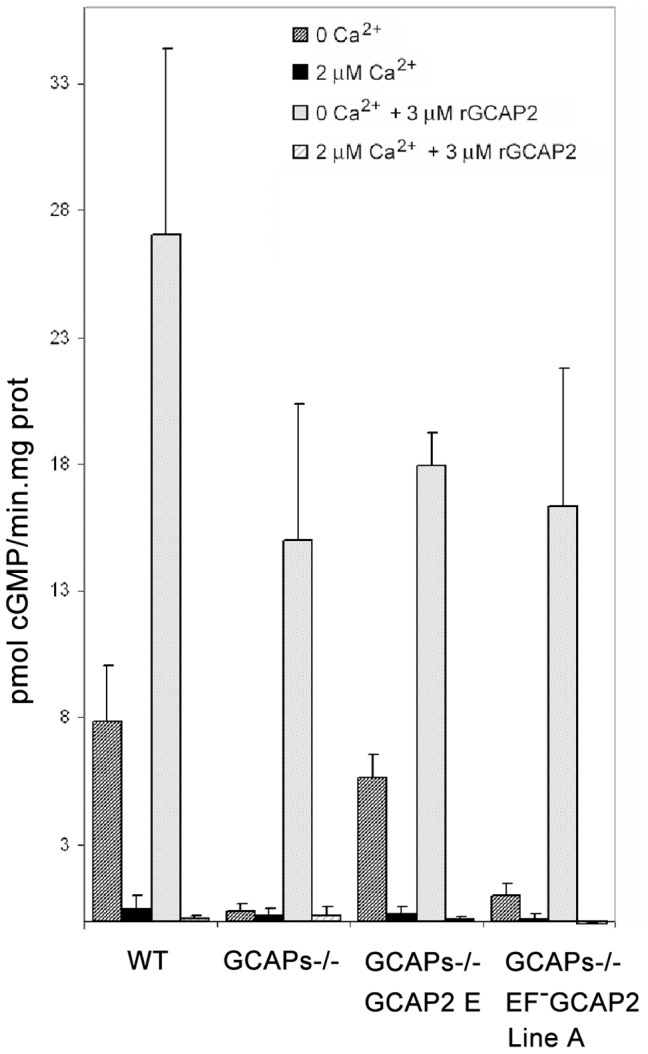
Guanylate cyclase activity in retinal homogenates of transgenic mice at 0 µM [Ca^2+^] and 2 µM [Ca^2+^]. Guanylate cyclase activity (pmol cGMP/min.mg prot) was determined in WT, GCAPs−/−, GCAPs−/− bGCAP2 line E and GCAPs−/− bEF^−^GCAP2 line A retinal extracts at 0 µM [Ca^2+^] or 2 µM [Ca^2+^] conditions, in the absence or presence of 3 µM recombinant GCAP2. In WT retinal homogenates at 0 µM [Ca^2+^] the endogenous GCAPs activate RetGC activity about 8-fold over the activity at 2 µM [Ca^2+^]. This stimulation of RetGC activity at 0 µM [Ca^2+^] is lost in GCAPs−/− retinal homogenates, but restored in the GCAPs−/− bGCAP2 line E, which indicates that the control bGCAP2 protein expressed *in vivo* as a transgene is active in these assays. However, retinal homogenates from GCAPs−/−bEF^−^GCAP2 line A mice showed greatly reduced RetGC activity at 0 µM [Ca^2+^] and no activity at 2 µM [Ca^2+^] conditions. Addition of 3 µM recombinant GCAP2 elicited activation of RetGC at 0 µM [Ca^2+^] in all retinal homogenates, indicating the presence of functional RetGC in all samples. These results show that bEF^−^GCAP2 was present, but mostly inactive, in GCAPs−/−bEF^−^GCAP2 line A retinal homogenates. Results show the mean and standard deviation of at least four independent experiments.

*In vitro* studies have shown that recombinant bEF^−^GCAP2 leads to maximal activation of Ret-GCs in reconstitution studies using washed bovine rod outer segment membrane preparations independently of free Ca^2+^ in the whole physiological range of [Ca^2+^] [38]. To assay whether the transgenic bEF^−^GCAP2 protein has the capacity to activate Ret-GC activity in retinal extracts from mice in a similar manner as in *in vitro* studies we performed guanylate cyclase activity assays in retinal extracts from the mutant or control mice obtained prior to significant retinal degeneration -between p20 and p30 - under conditions of 0 Ca^2+^ or 2 µM Ca^2+^ (Fig. 3). Ca^2+^-dependent modulation of Ret-GC activity was observed in retinal homogenates from wildtype mice and control GCAPs−/− bGCAP2 E line. As expected, the Ca^2+^-sensitive guanylate cyclase activity was undetectable in GCAPs−/− retinal extracts, indicating that the guanylate cyclase activity that is measurable in whole mouse retinal extracts originates essentially from photoreceptor cells in a GCAPs-dependent manner. As a control for the presence of functional Ret-GCs in retinal extracts, guanylate cyclase activity was also measured after addition of 3 µM recombinant bGCAP2, which restored robust activity in a Ca^2+^ dependent manner. Surprisingly, retinal extracts from GCAPs−/− bEF^−^GCAP2 B mice resembled those of GCAPs−/−. They showed little detectable retGC activity at either 0 Ca^2+^ or high Ca^2+^. Even though the levels of Ret-GCs and bEF^−^GCAP2 were reduced to some extent in these retinal extracts due to the shortening of the rod outer segments in this line, the addition of recombinant bGCAP2 showed that there was functional Ret-GCs in these extracts at levels that were sufficient to elicit a measurable activity. The results shown are the average of four independent experiments. These results indicate that while the transgenic bGCAP2 control protein expressed in the GCAPs−/− background reproduced normal activity, the transgenic mutant form of bGCAP2 impaired to bind Ca^2+^ showed very little detectable activity *in vivo*.

**Figure 4:**
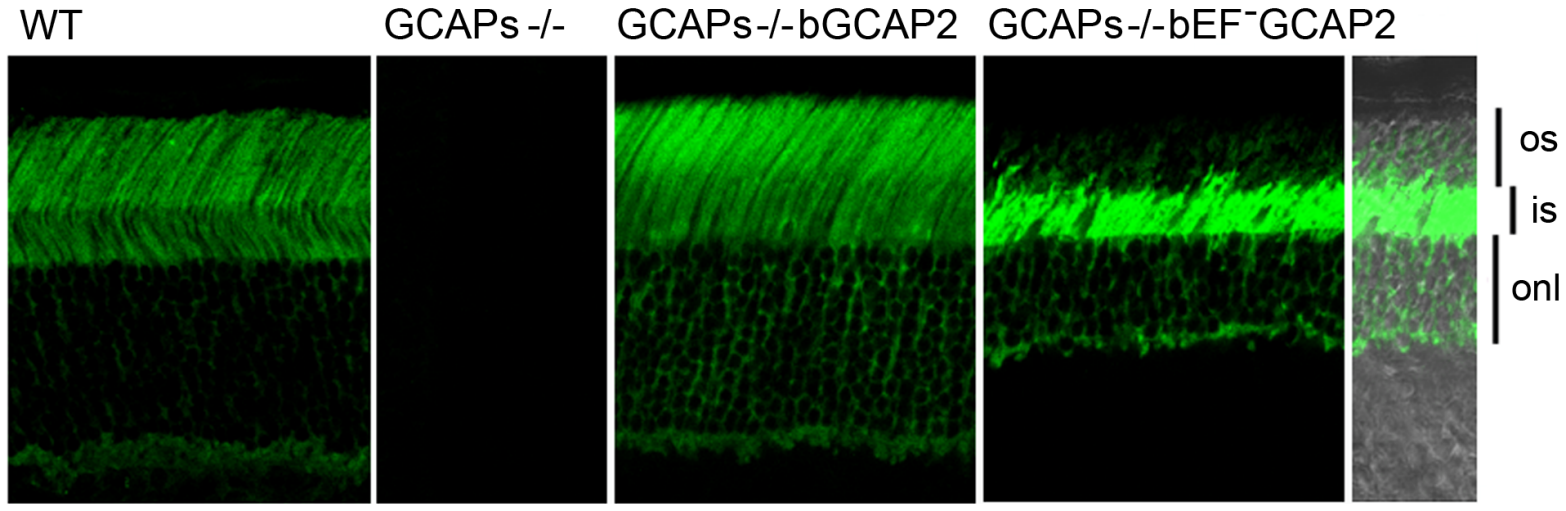
bEF^−^GCAP2 mislocalizes in transgenic retinas, accumulating at the inner segment compartment of the cell. Cryosections of central retina from WT, GCAPs−/−, GCAPs−/− bGCAP2 line E and GCAPs−/− bEF^−^GCAP2 line B, immunostained with an anti-GCAP2 polyclonal Ab. Endogenous GCAP2 in WT retinas distributes to the cytosolic space of rod cells, at the rod outer segment, inner segment, outer nuclear and outer plexiform layers of the retina. This pattern of staining is lost in GCAPs−/− retinas and restored in GCAPs−/−bGCAP2 line E retinas. However, in GCAPs−/− bEF^−^GCAP2 line B the pattern of staining is shifted, with the GCAP2 signal being much stronger at the inner segment and proximal compartments of the cell than at the outer segment. *os*, outer segment; *is*, inner segment; *onl*, outer nuclear layer.

To study whether the bEF^−^GCAP2 protein reproduced the localization pattern of endogenous GCAP2 in transgenic mice, we immunostained GCAP2 in retinal cryosections. Whereas transgenic bGCAP2 in the control line mimicked the localization of endogenous GCAP2 in wildtype retinas (staining the outer segment, inner segment, cytosol of outer nuclear layer and outer plexiform layers of the retina, with the signal being most intense at rod outer segments); this pattern was shifted in the case of bEF^−^GCAP2, with the signal being most intense at the rod proximal compartments, particularly at the inner segment layer (Fig. 4). These results show that bEF^−^GCAP2, when expressed in the GCAPs−/− background, tend to accumulate at the metabolic compartment of the cell.

These results indicate that bEF^−^GCAP2 in the retinas of transgenic mice has a greatly reduced capacity to activate the cyclase and accumulates at the inner segment of the cell, indicating that the pathology in these mice does not result from unabated cGMP synthesis. Furthermore, the retinal degeneration in bEF^−^GCAP2 mice could not be prevented by raising the mice in constant light exposure that would counteract the increase in cGMP synthesis by continuous cGMP hydrolysis (Fig. S3), as was the case in Y99C-GCAP1 mice [26].

Taken together, these results point to a mechanism independent of cGMP metabolism as the molecular basis for the neurodegeneration in these mice.

### bEF^−^GCAP2 protein is phosphorylated to high levels *in vivo* and binds to 14-3-3 in a phosphorylation-dependent manner

We reasoned that the accumulation of bEF^−^GCAP2 at the proximal compartments of the cell rather than its absence at the rod outer segment was the cause of the progressive retinal degeneration in these mice, given that the absence of GCAP1 and GCAP2 in GCAPs−/− mice does not affect gross retinal morphology [11]. To address why bEF^−^GCAP2 fails to be distributed to the rod outer segment and how its retention and accumulation at the inner segment leads to toxicity, we investigated the protein-protein interactions that the mutant form of the protein establishes in a specific manner. Immunoprecipitation assays were conducted with an anti-GCAP2 monoclonal antibody cross-linked to magnetic beads, using Triton X100-solubilized whole retinal extracts from GCAPs−/− bGCAP2 E and GCAPs−/− bEF^−^GCAP2 B mice. Retinal extracts from GCAPs−/− mice were carried to define the background. The pool of proteins immunoprecipitated in each case was identified by directly subjecting the elution fractions to trypsin-digestion and liquid chromatography-tandem mass spectrometry analysis (LC-MS/MS). We searched for proteins identified in the GCAPs−/− bEF^−^GCAP2 B sample with an spectral counting at least 1.5-fold over the GCAPs−/− bGCAP2 and GCAPs−/− control lines). We found that only the distinct isoforms of 14-3-3 proteins fulfilled these criteria, being identified with a considerably higher number of peptides [1.33 to 3.2-fold higher] in the GCAPs−/−bEF^−^GCAP2 B than in control samples in at least two independent experiments (Table 1). Spectral counting of 14-3-3 isoforms were between 1.6-fold and 5-fold higher in the GCAPs−/−bEF^−^GCAP2 B samples than in control samples in the two experiments (Table S1).

**Table 1:** Proteins identified by LC-MS/MS in GCAP2 immunoprecipitation experiments.

				Exp 1			Exp 2		
Protein	UniProtKB/Swiss-Prot entry name	Primary accession number	Gene name	line E	line B	ctrl	line E	line B	ctrl
GCAP 2	GUC1B-BOVIN	P51177	GUCA1B	12	10	0	9	6	0
14-3-3 protein ε	1433E-MOUSE	P62259	Ywhae	11	19	6	5	10	5
14-3-3 protein γ	1433G-MOUSE	P61982	Ywhag	9	12	4	4	8	3
14-3-3 protein ζ/δ	1433Z-MOUSE	P63101	Ywhaz	7	15	9	6	9	6
14-3-3 protein β/α	1433B-MOUSE	Q9CQV8	Ywhab	6	13	6	0	8	0
14-3-3 protein τ	1433T-MOUSE	P68254	Ywhaq	5	16	5	3	7	3

The table lists proteins identified in GCAP2 immunoprecipitation assays from retinal homogenates of GCAPs−/− bGCAP2 line E, GCAPs−/− bEF^−^GCAP2 line B and GCAPs−/− mice (ctrl). Data is shown from two independent experiments (three columns per experiment). Last six columns indicate the number of peptides identified for each protein in each sample, indicative of the relative levels of coimmunoprecipitated proteins. GCAP2 was immunoprecipitated to similar levels in control line E and mutant line B, but 14-3-3 isoforms coimmunoprecipitation with GCAP2 occurred substantially more efficiently in mutant line B than in control line E, indicating 14-3-3 selective binding to GCAP2 locked in its Ca^2+^-free form. GCAP2, guanylate cyclase activating protein 2.

**Figure 5:**
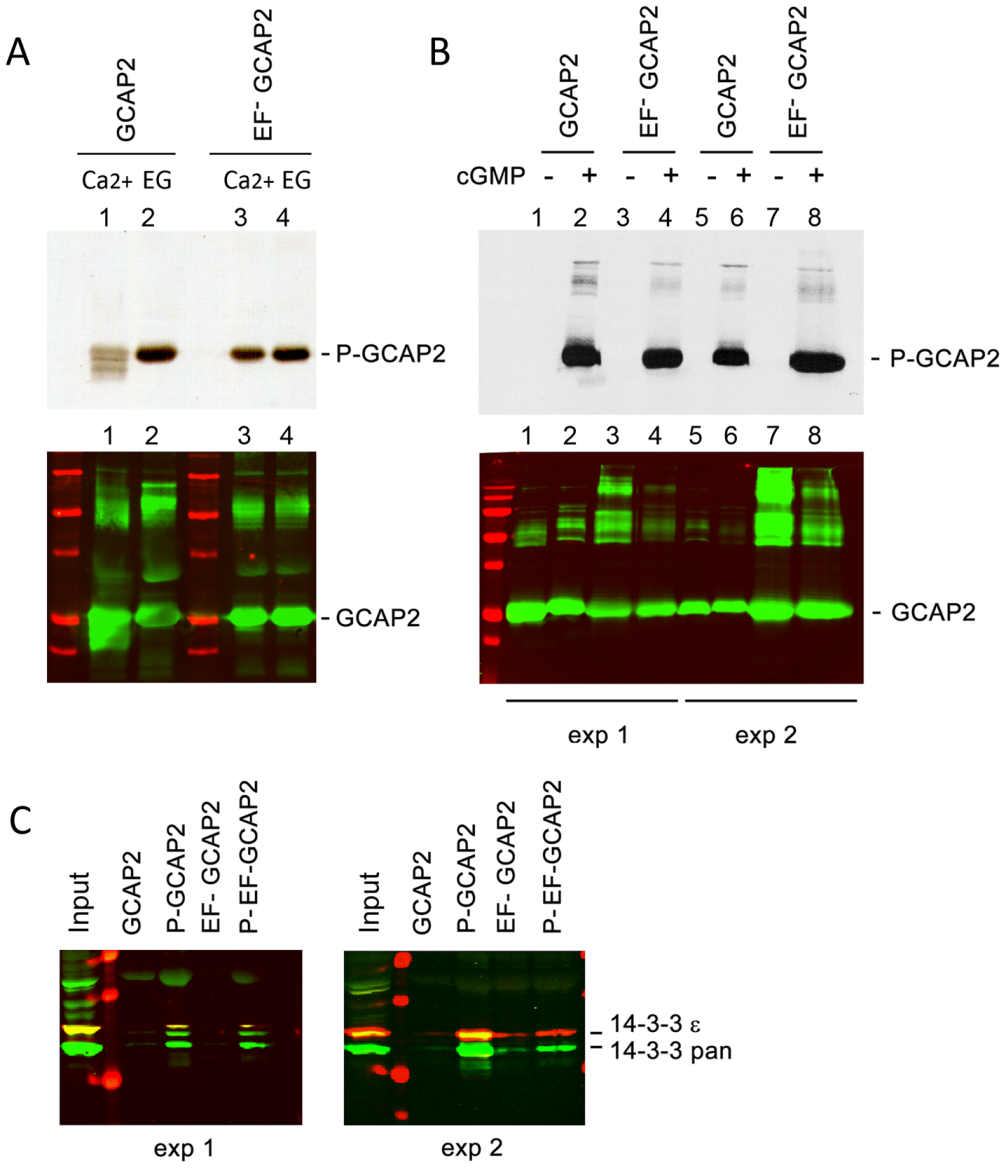
The protein 14-3-3 binds to recombinant GCAP2 in a phosphorylation-dependent manner. **A.** *In vitro*, Ca^2+^-free bGCAP2 is phosphorylated more efficiently than Ca^2+^-bound bGCAP2. Upper panel shows an autoradiograph of ^33^P phosphorylation products from an *in vitro* phosphorylation reaction of recombinant wildtype bGCAP2 or bEF^−^GCAP2 with protein kinase G (PKG), in the presence or absence of free Ca^2+^. The 20 µl reaction mixture contained 8.5 µg of purified recombinant wildtype bGCAP2 or bEF^−^GCAP2, purified PKGIα (100 units, Calbiochem) and 3 µCi of ^33^P- γATP in phosphorylation reaction buffer, containing either CaCl_2_ or EGTA (see Methods). After incubation, reaction mixtures were resolved by 15% SDS-PAGE and transferred to a nitrocellulose membrane. Lower panel shows immunostained GCAP2. Recombinant bGCAP2 or bEF^−^GCAP2 protein were present to similar amounts in all reaction tubes. **B.** *In vitro* phosphorylated or mock-treated bGCAP2 and bEF^−^GCAP2 were generated for pull-down assays. Phosphorylation reactions were performed as above, in the presence of EGTA, except that cGMP was added to 500 µM (+ lanes) or not added (− lanes). Immunostaining of GCAP2 in the same nitrocellulose membrane shows the GCAP2 monomer at 25 kDa and upper bands corresponding to dimers and multimers of GCAP2, observed to a higher extent in the EF^−^GCAP2 lanes. Molecular mass (MW) markers (Precision Plus Protein Standards, BioRad) are 20, 25, 37, 50, 75, 100 and 150 kDa. Experiment shown in duplicate. **C.** The 14-3-3 protein isoforms bind more efficiently to phosphorylated bGCAP2 and bEF^−^GCAP2 than to unphosphorylated counterparts. Phosphorylated or mock- proteins were cross-liked to magnetic beads and pull-down assays were performed with whole bovine retinal extracts obtained in 1% Triton-X100. Panels show the input and bound fractions for the indicated phospho- or mock-proteins, resolved by 15% SDS-PAGE. Membrane was sequentially incubated with a pAb to 14-3-3pan (IBL International, Hamburg, Germany), a mAb to 14-3-3ε (abcam, Cambridge, UK), an IRDye 800CW Goat Anti-rabbit IgG and a IRDye 680CW Goat Anti-mouse IgG (Tebu-Bio, Offenbach, Germany). Image was acquired at the Odyssey Imaging System (LI-COR). Therefore 14-3-3pan isoforms (30 kDa) are shown in green, while 14-3-3ε (33 kDa) is shown in red. Experiment shown in duplicate.

Because 14-3-3 proteins typically bind to their targets in response to phosphorylation [39], and since phosphorylation of GCAP2 has been reported to occur *in vitro* at a conserved Ser at position 201 in bGCAP2 [30], we next assayed whether the binding of 14-3-3 to GCAP2 was phosphorylation dependent. We first reproduced the observation that GCAP2 can be phosphorylated *in vitro* by PKG, with Ca^2+^-free bGCAP2 being a better substrate for the kinase than Ca^2+^-loaded bGCAP2 (Fig. 5A). Subsequently, we used recombinant bGCAP2 or bEF^−^GCAP2 in *in vitro* phosphorylation reactions with PKG to generate phosphorylated-bGCAP2 or mock-treated bGCAP2 for pull-down assays with bovine whole retinal homogenates (Fig. 5B). As seen in Fig. 5C, 14-3-3 showed preferential binding to the phosphorylated form of bGCAP2 or bEF^−^GCAP2 in two independent experiments.

The observations that 14-3-3 binds more efficiently to bEF^−^GCAP2 than to bGCAP2 *in vivo* and that 14-3-3 binds to bGCAP2 in a phosphorylation dependent manner, together with the reported higher efficiency of GCAP2 phosphorylation in its Ca^2+^-free rather than its Ca^2+^-bound conformational state led us to hypothesize that bEF^−^GCAP2 might be abnormally phosphorylated in the living cell. To test this hypothesis we performed a ^32^P_i_ -metabolic labeling of GCAPs−/− bGCAP2 and GCAPs−/− bEF^−^GCAP2 retinas *in situ*, followed by GCAP2 immunoprecipitation and SDS-PAGE analysis. Following the incorporation of ^32^P_i_ into the retinas of dark-adapted mice for 2 h, retinas were either kept in darkness or exposed to 5 min of bright white light and immediately subjected to Triton X100-solubilization and GCAP2 immunoprecipitation. GCAPs−/− retinas were carried as a negative control.

**Figure 6:**
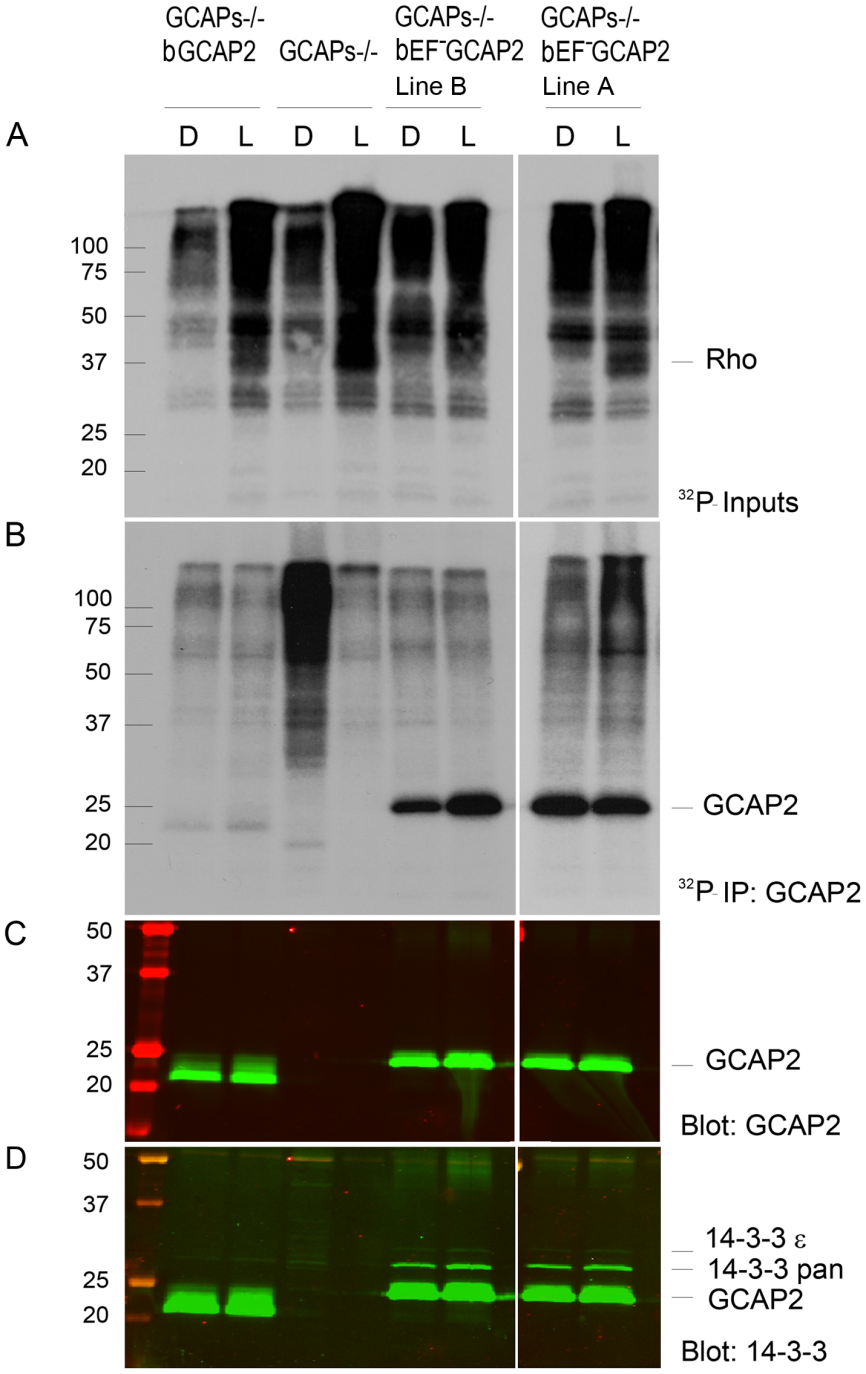
^32^P_i_ metabolic labeling reveals phosphorylation of bEF^−^GCAP2 to a higher extent than bGCAP2 in living retinas. **A.** *In situ* phosphorylation assay. Retinas from dark-adapted mice from the indicated phenotypes were dissected under dim red light, incubated in bicarbonate buffered Locke's solution containing 1 mCi/mL of ^32^P-H_3_PO_4_ for 90 min in a 5% CO_2_ incubator and exposed to white light for 5 min (L) or maintained in darkness (D). Retinas were homogenized in Triton X100-solubilization buffer and pre-cleared by centrifugation. Aliquots corresponding to one tenth of a retina were resolved by 15% SDS-PAGE and blotted to a nitrocellulose membrane. Phosphorylated proteins were visualized by autoradiography upon 4 h of exposure. **B.** GCAP2 immunoprecipitation in ^32^P-labeled samples. Solubilized samples corresponding to two retinas per phenotype and condition were used as inputs for GCAP2 immunoprecipitation with an anti-GCAP2 mAb. Elution fractions were resolved by 15% SDS-PAGE, blotted to a nitrocellulose membrane and visualized by autoradiography after 4 days of exposure. **C.** Western blot of samples in (B) using a polyclonal antibody anti-GCAP2 show that the amount of GCAP2 in the control GCAPs−/− bGCAP2 E line was comparable to that in mutant lines GCAPs−/− bEF^−^GCAP2 A and B. **D.** Immunostaining of 14-3-3 proteins in the same membrane, by using a pAb to 14-3-3pan (IBL International, Hamburg, Germany).

Fig. 6A shows equal fractions of the Triton X100-solubilized retinas after ^32^P_i_-incorporation and 5 min dark- or light-exposure. The overall pattern of bands in this panel shows that incorporation of ^32^P_i_ into the ATP pool of the retina occurred at comparable levels in all samples, allowing the detection of phosphorylated proteins and changes in the overall phosphorylation pattern caused by light (e.g. the light-dependent phosphorylation of rhodopsin is observed at 35–37 kDa). GCAP2 phosphorylation could not be detected in whole retinal extracts, so these samples were used as inputs for the GCAP2 immunoprecipitation assay shown in Fig. 6B. GCAP2 was phosphorylated to low levels in the GCAPs−/− bGCAP2 sample in the dark, and to a slightly higher extent when the retina was exposed to light. No 24 kDa bands were observed in the GCAPs−/− samples. Strikingly high levels of bEF^−^GCAP2 phosphorylation were observed in GCAPs−/− bEF^−^GCAP2 samples (lines A and B). A GCAP2 immunoblot of the ^32^P-labeled membrane confirmed that comparable levels of GCAP2 were immunoprecipitated in GCAPs−/− bGCAP2 and GCAPs−/− bEF^−^GCAP2 samples (Fig. 6C). Fig. 6D shows the subsequent immunostaining of the 14-3-3 pan and epsilon isoforms in the same membrane, further confirming the selective binding of 14-3-3 to the phosphorylated mutant form of GCAP2 impaired to bind Ca^2+^.

**Figure 7:**
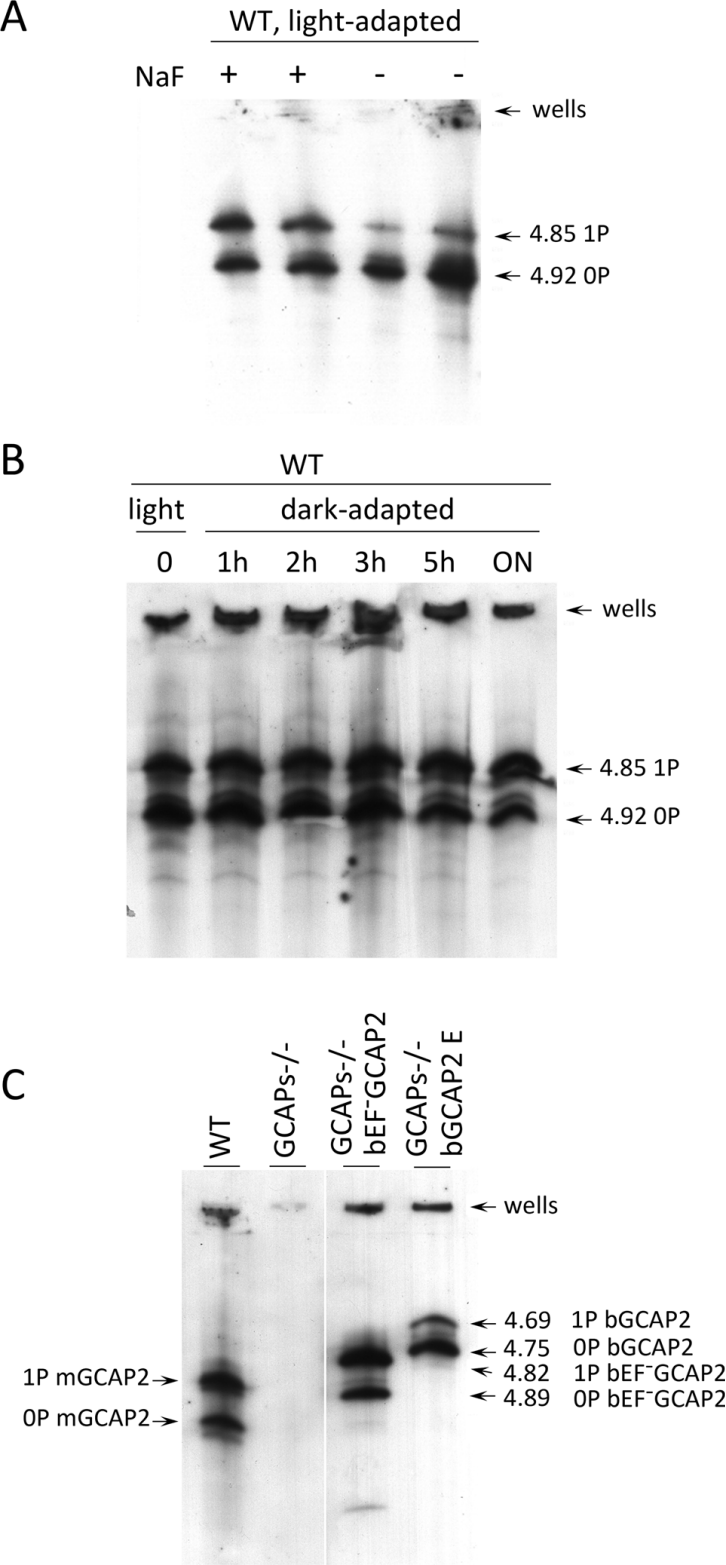
Analysis of GCAP2 phosphorylation by isoelectrofocusing. **A**. Isoelectrofocusing (IEF) gel of light-adapted wildtype mouse retinal homogenates. Mice were light-adapted to room light. Retinas were obtained, solubilized in a saline buffer with 1% dodecyl maltoside, in the presence or absence of 50 mM NaF (phosphatase inhibitor). Samples were clarified, loaded onto an electrofocusing gel (pH range 3–8) and focused for 2 h at 23W. Proteins were transferred to a nitrocellulose membrane and incubated with an anti-GCAP2 Ab. Two prominent bands are observed at 4.92 and 4.85 isoelectric point, that correspond to unphosphorylated and monophosphorylated mGCAP2, respectively. **B**. The overall phosphorylation status of GCAP2 does not change significantly during a 12 h period of dark-adaptation. Mice were light-adapted to room light for 1 h, and subjected to dark-adaptation for a period of up to 14 h. Retinas were analyzed as above. **C**. Analysis of GCAP2 phosphorylation status in the indicated mouse lines. Transgenic bGCAP2 is phosphorylated to a lesser extent than the endogenous mGCAP2, whereas bEF^−^GCAP2 is phosphorylated to a much higher extent. Note that the isoelectric point of bGCAP2 differs from that of mGCAP2, and that the isoelectric point of bEF^−^GCAP2 (E80Q,E116Q,D158N GCAP2) is shifted versus that of bGCAP2. Results from the isoelectrofocusing gels confirm that transgenic bEF^−^GCAP2 is phosphorylated to a much higher extent than the control transgenic bGCAP2.

GCAP2 phosphorylation was further characterized by isoelectrofocusing gel analysis followed by immunoblotting with a GCAP2 antibody (Fig. 7). Under room light conditions wildtype C57/B6 mice showed two bands of roughly equal intensity corresponding to the pI of the unphosphorylated (4.92) and singly phosphorylated (4.85) mGCAP2. The intensity of the 4.85 band was greatly diminished when NaF, a broad phosphatase inhibitor, was omitted from the samples, thus confirming the identity of this band as phosphorylated GCAP2 (Fig. 7A). We conclude that about half of the total GCAP2 protein is phosphorylated in wildtype mice under standard room light conditions. The extent to which endogenous mGCAP2 was phosphorylated in wildtype mice under room light conditions was higher than that of bGCAP2 in GCAPs−/− bGCAP2 transgenic mice.

To address whether GCAP2 phosphorylation takes place differentially in dark/light conditions, wildtype mice that were adapted to room light for 1 h were dark-adapted for up to 14 h, and GCAP2 phosphorylation was analyzed at 1, 2, 3, 5 and 14 h. Fig. 7B shows that the ratio of unphosphorylated to phosphorylated GCAP2 did not vary substantially during the 14 h dark-adaptation period. If we presume that GCAP2 is preferentially phosphorylated during periods of light exposure when in its Ca^2+^-free conformation, these results may indicate that a few hours of dark- or light-adaptation are not enough to have a noticeable effect on the overall GCAP2 population. This would not be surprising if only newly synthesized GCAP2 was subjected to the kinase/phosphatase regulation (see Discussion).

Isoelectrofocusing of retinal samples from GCAPs−/− bEF^−^GCAP2 and GCAPs−/− bGCAP2 were performed to assay the steady-state relative levels of non-phosphorylated and phosphorylated GCAP2 (Fig. 7C). Whereas endogenous GCAP2 in wildtype C57/B6 mice showed similar proportions of non-phosphorylated and phosphorylated GCAP2, the GCAPs−/− bEF^−^GCAP2 sample showed a larger fraction of phosphorylated GCAP2 and the GCAPs−/− bGCAP2 sample showed the reverse: a larger fraction of non-phosphorylated GCAP2. These results are consistent with the metabolic labeling results, namely, low levels of phosphorylation in the GCAPs−/− bGCAP2 control line, and much higher phosphorylation levels in the GCAPs−/− bEF^−^GCAP2 line (Fig. 7C and Fig. 6B).

**Figure 8:**
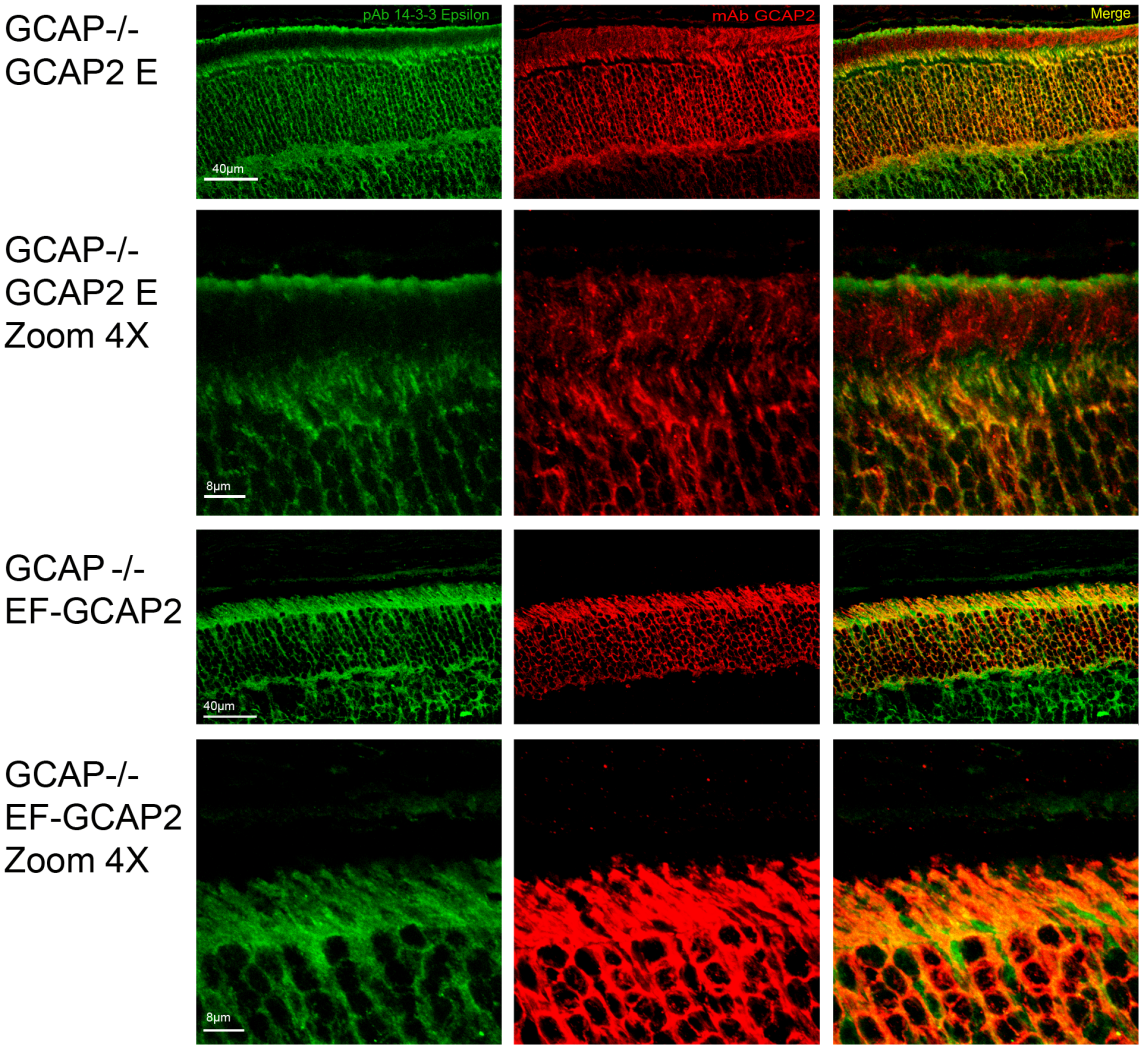
Coimmunolocalization of 14-3-3ε with GCAP2 in retinas from GCAPs−/−bGCAP2 E and GCAPs−/− bEF^−^GCAP2 B mice. Cryosections of central retina from the indicated lines at 20 days of age were immunostained with an anti-GCAP2 mAb (Affinity Bioreagents, Golden, Colorado, USA) and an anti-14-3-3ε rabbit monoclonal (abcam, Cambridge, UK), by indirect immunofluorescence staining with the Alexa 488 goat anti-rabbit IgG and Alexa 555 goat anti-mouse IgG (Molecular Probes, Eugene, Oregon). GCAP2 signal in red, 14-3-3ε signal in green. GCAP2 and 14-3-3 proteins colocalize at the inner segment and proximal compartments of photoreceptor cells.

To address whether 14-3-3 binding to phosphorylated GCAP2 might be the cause of its retention at inner segments, we analyzed the localization of the 14-3-3 proteins in retinal sections from GCAPs−/− bGCAP2 and GCAPs−/− bEF^−^GCAP2 samples. Fig. 8 shows that 14-3-3 epsilon localizes to all cell layers of the retina; the ganglion cell layer, the inner cell layer and the photoreceptor cell layer of the retina. In photoreceptor cells it appears to distribute to the inner segment, the perinuclear region and the synaptic terminal, but it is excluded from the outer segment. This isoform of 14-3-3 colocalized with GCAP2 mainly at the inner segment of GCAPs−/− bGCAP2 samples, but also to the perinuclear region and synaptic terminals in the GCAPs−/− bEF^−^GCAP2 samples. From these results we infer that the localization pattern of 14-3-3ε in photoreceptor cells would be consistent with a role of GCAP2 phosphorylation and 14-3-3 binding at retaining the mutant form of GCAP2 impaired to bind Ca^2+^ at the proximal compartments of the cell.

### Phosphorylation at Ser201 is required for the retention of bEF^−^GCAP2 at the proximal compartments of the photoreceptor cell *in vivo*

To address whether phosphorylation of GCAP2 is what causes the retention of bEF^−^GCAP2 at the inner segment and proximal compartments of the cell, we expressed a mutant form of bEF^−^GCAP2 in which Ser201 was mutated to Gly as a transient transgene in rod cells, given that Ser201 is the only residue that was found to be phosphorylated in GCAP2 [30].

The bS201G/EF^−^GCAP2 cDNA was expressed under the rod opsin promoter by subretinal injection and *in vivo* electroporation of the DNA in neonatal GCAPs−/− mice as described [40]. Both bGCAP2 and bEF^−^GCAP2 cDNAs were carried out in parallel in order to compare the localization of the mutants under equivalent experimental conditions. A plasmid expressing the green fluorescent protein (GFP) under the Ubiquitin C promoter was coinjected to identify the region around the injection site in which DNA transfection was efficient, and electroporated retinas were analyzed at p28.

**Figure 9:**
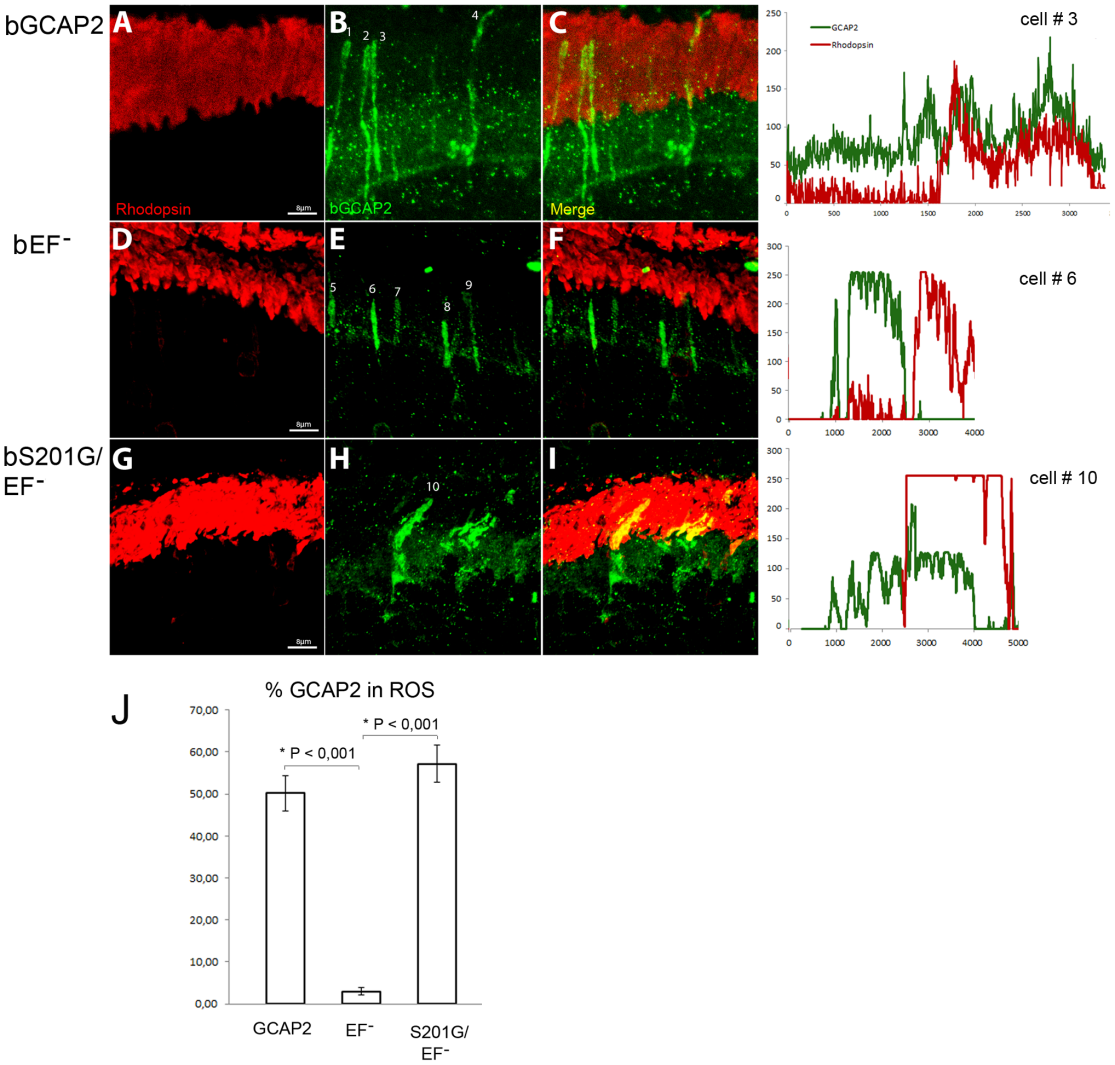
Mutation of Ser201 in bEF^−^GCAP2 precludes protein retention at the inner segment. Wildtype bGCAP2, bEF^−^GCAP2 or bS201G/EF^−^GCAP2 were transiently expressed in the rod photoreceptor cells of GCAPs−/− mice by *in vivo* DNA electroporation of neonates following subretinal injection. A plasmid expressing GFP driven by the Ubiquitin C promoter was coinjected in order to identify transfected areas in the eye at postnatal day 28 (p28). Cryosections were immunostained for GCAP2 with a polyclonal GCAP2 Ab and an Alexa Fluor 555 anti-rabbit IgG (signal converted to green); and for rhodopsin with monoclonal Ab 1D4 and an Alexa Fluor 647 anti-mouse IgG (red signal). Transient expression of wildtype bGCAP2 by electroporation reproduced the reported localization pattern in the stable transgenic line, namely, its almost equal distribution between the inner and outer segments (panels **A–C**, profile for cell N.3). bEF^−^GCAP2 was mostly retained at the inner segments, being excluded from outer segments (panels **D–F**, and profile for cell N.6), which also reproduced the observation from the stable bEF^−^GCAP2 transgenic lines. In contrast, bS201G/EF^−^GCAP2 localized to some extent at the inner segment but mostly distributed to rod outer segments, showing a clear colocalization with rhodopsin (panels **G–I**, and profile from cell N.10). Panel **J** shows a histogram of the mean percentage of GCAP2 distribution to ROS for each phenotype and the standard error of the mean, as determined by calculating: (the intensity of GCAP2 that colocalizes with rhodopsin)/(intensity of GCAP2 that colocalizes with rhodopsin + intensity of GCAP2 at the inner segment), see Methods. (Five cells were analyzed for the WT sample, twelve cells for bEF^−^GCAP2 and thirteen cells for bS201G/GCAP2).

Fig. 9 shows that the localization of bGCAP2 and bEF^−^GCAP2 in the transient transgenic mice obtained by electroporation reproduced the localization observed in stable transgenics: specifically, bEF^−^GCAP2 was retained at the inner segment and proximal compartments of transfected photoreceptors. bEF^−^GCAP2 was excluded from the outer segment, which is demarcated by rhodopsin immunofluorescence (red) (Fig. 9 panels E, F and profile from cell N.6, Fig. S4 for additional images and profiles). In contrast, the mutant bS201G/EF^−^GCAP2 distributed to the proximal compartments of the cell but also to rod outer segments. As shown in panels H-I of Fig. 9 and in the profile from cell N. 10, the GCAP2 signal -in green- co-labeled with rhodopsin (red) in all transfected cells (thirteen cells analyzed, 57% of GCAP2 signal co-labeled with rhodopsin on average, see Fig. S4), indicating its redistribution to rod outer segments. On average, 50% of the protein distributed to rod outer segments when bGCAP2 was expressed, whereas virtually all bEF^−^GCAP2 was retained at the inner segment and proximal compartments. Mutating S201 to Gly in bEF^−^GCAP2 reverted this retention, resulting in 57% of the protein distributing to rod outer segment (histogram in Fig. 9J).

These results indicate that phosphorylation at Ser201 in the mutant form of GCAP2 impaired to bind Ca^2+^ is what causes its accumulation at the inner segment and proximal compartments of the cell, ultimately leading to toxicity.

### Light conditions expected to promote a sustained reduction in the concentration of Ca^2+^ at the inner segment compartment lead to GCAP2 retention at the inner segment in wildtype mice

The finding that GCAP2 locked in its Ca^2+^-free form (bEF^−^GCAP2) is retained at the inner segment compartment resulting in toxicity could have important implications for disease, if it meant that endogenous Ca^2+^-free GCAP2 would be retained at the inner segment in wildtype mice during conditions that promoted a sustained reduction in [Ca^2+^]_i_. Mutations in several genes involved in the light response result in the sustained hyperpolarization of the cell and a steady reduction in [Ca^2+^]_i_. Null mutations in GUCY2D or RD3 causative of the Lebers Congenital Amaurosis (LCA) form of blindness, for instance, would result in reduced levels of cGMP at the rod outer segments, the closure of cGMP-channels and hyperpolarization of the cell, with the ensuing reduction in the influx of Ca^2+^ to rod inner segment [36], [37], [41]. Also associated to LCA, null mutations in RPE65 result in retinal degeneration due to the basal constitutive activity associated to opsin, the apoprotein form of the visual pigment, leading to a sustained hyperpolarization of the cell [42]. These genetic disorders that ultimately cause an effect similar to continuous light exposure, are collectively referred to as “equivalent-light” disorders [43]. Therefore, if GCAP2 was retained at the inner segment under constant illumination conditions, it would be an indication that this pathway could contribute to the pathology of these disorders.

**Figure 10:**
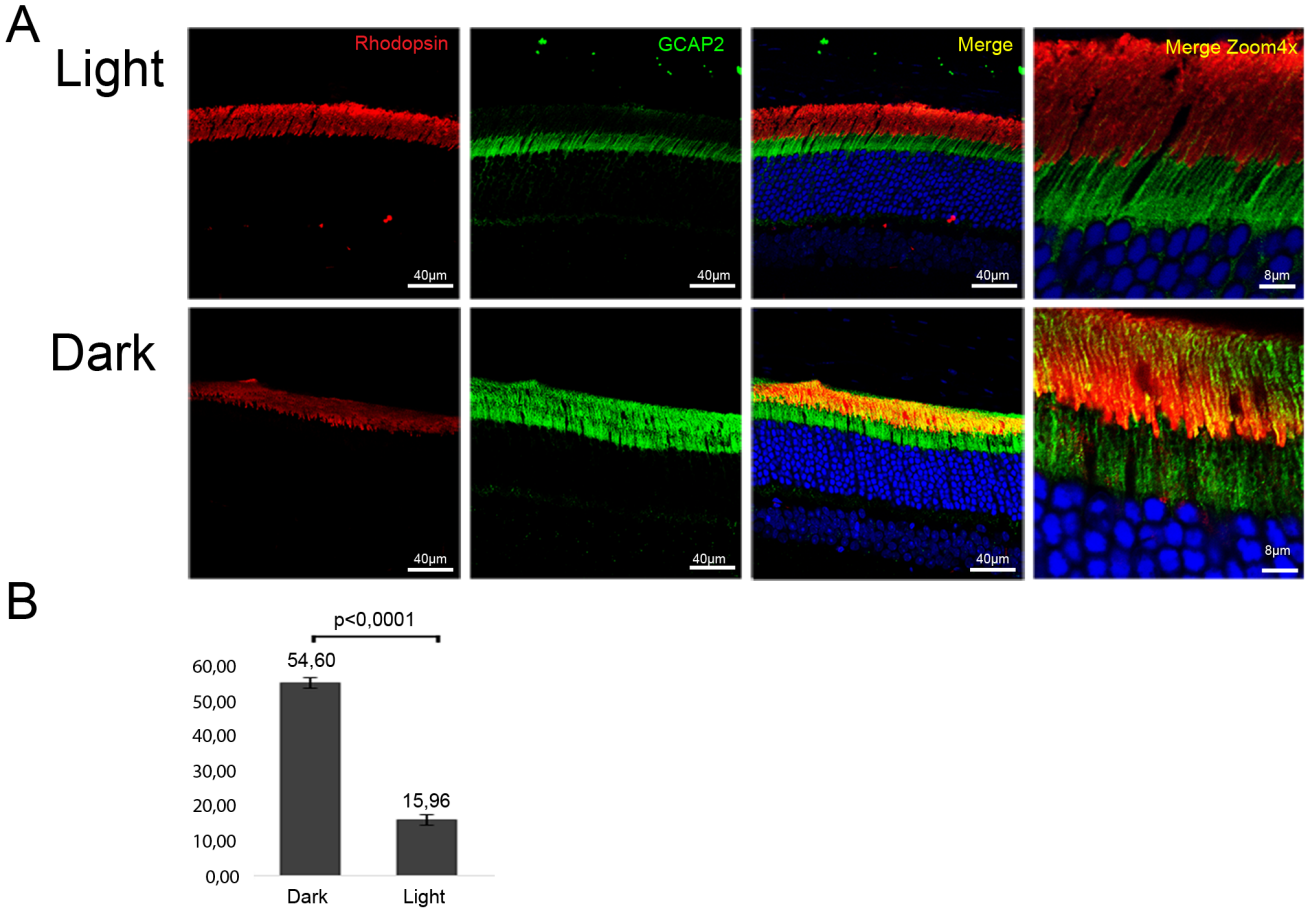
Constant light exposure for 30 days retains endogenous GCAP2 at the inner segment in wildtype mice. **A.** Wildtype mice (C57Bl/6J) were exposed to white fluorescent light of 700 lux intensity for 30 days (upper panel row), or kept in constant darkness for 30 days (lower row). Cryosections of central retina were immunostained with an anti-GCAP2 pAb and an anti-rhodopsin mAb, by indirect immunofluorescence with Alexa 488 goat anti-rabbit IgG and Alexa 555 goat anti-mouse IgG (molecular Probes, Oregon). GCAP2 signal in green, rhodopsin signal in red. **B.** Z-axis stacks were obtained from four mice per condition, and the percentage of GCAP2 signal colocalizing with rhodopsin at the rod outer segment layer was quantified by ImageJ (see Methods). Values for the percentage of GCAP2 co-localizing with rhodopsin were 54,60%±1,47 (n = 4) in dark samples, and 15,96%±2,05 (n = 4) in light samples. Student T-test P<0,001 (GraphPad Prism 6).

Wildtype mice were exposed to constant fluorescent light (700 lux), or kept in dark cabinets for thirty days, and GCAP2 subcellular distribution was analyzed by immunofluorescence (Fig. 10). The percentage of GCAP2 co-localizing with rhodopsin was quantified in four mice per condition. After 30 days of light exposure 84% of GCAP2 was retained at the inner segment, in contrast to 45% of GCAP2 retained in mice kept for 30 d in constant darkness, P≤0,0001 (histogram Fig. 10).

These results show that a sustained drop in [Ca^2+^]_i_ in wildtype rod photoreceptors causes GCAP2 retention at the inner segment. This, together with the results obtained from bEF^−^GCAP2 transgenic mice showing that accumulation of bEF^−^GCAP2 at the inner segment results in toxicity, points to the sustained retention of Ca^2+^-free GCAP2 at the inner segment as a pathway that could contribute to the pathology of “light-equivalent” disorders.

## Discussion

### Precluding Ca^2+^ binding to GCAP2 *in vivo* leads to retinal degeneration

We here report that a form of GCAP2 with mutations that impair Ca^2+^ coordination at the three functional EF-loops (bEF^−^GCAP2) led to retinal degeneration when expressed in rods in transgenic mice. *In vitro* the bEF^−^GCAP2 mutant shows a similar shift in Ca^2+^ sensitivity of guanylate cyclase regulation as the Y99C, E155G and other GCAP1 mutants that directly or indirectly affect Ca^2+^ coordination [22], [24], [38]. These GCAP1 mutants have been demonstrated to cause retinal degeneration *in vivo* by leading to persistent activation of the cyclase, causing elevated levels of cGMP and Ca^2+^ [23], [25], [26]. Intriguingly, we found that the retinal degeneration caused by bEF^−^GCAP2 expression in rods was independent of cGMP metabolism. When guanylate cyclase activity was measured in retinal homogenates from bEF^−^GCAP2 transgenic mice, instead of constitutive activation of the cyclase we found very diminished cyclase activity independently of the [Ca^2+^] conditions, which contrasts with the normal cyclase activity observed in homogenates of wildtype and bGCAP2 control-transgenic mice (Fig. 3). Furthermore, retinal degeneration in bEF^−^GCAP2 transgenic mice could not be prevented or delayed by raising the mice under constant light exposure (Fig. S3). These results show for the first time that functional EF-hands in GCAP2 are required for photoreceptor cell integrity *in vivo*, by a mechanism independent of guanylate cyclase regulation.

### Phosphorylation of GCAP2 and 14-3-3 binding as a new *in vivo* mechanism controlling GCAP2 subcellular distribution that causes toxicity when overly deregulated

In contrast to the bGCAP2 control-transgenic protein that reproduced the endogenous mGCAP2 subcellular localization, bEF^−^GCAP2 largely accumulated at inner segment and proximal compartments of the rod when it was expressed in the GCAPs−/− background (Fig. 4). At this compartment, bEF^−^GCAP2 was phosphorylated to a much higher extent than the control transgenic protein in *in situ* phosphorylation assays as well as under steady state conditions in the intact rod as shown by IEF, and it was found to bind 14-3-3 proteins (Table 1, Figs. 5–7). This constitutes the first report that GCAP2 is phosphorylated *in vivo*, at much higher levels when locked in its Ca^2+^-free conformation, and that phosphorylation of GCAP2 triggers 14-3-3 binding. We show that 14-3-3 localization in rod photoreceptors is restricted to proximal compartments and excluded from the outer segments (Fig. 8). Furthermore, we demonstrate that phosphorylation is required for bEF^−^GCAP2 retention at proximal compartments by showing that replacing Ser201 by Gly in bEF^−^GCAP2 substantially reverts this retention (Fig. 9). On average 57% of bS201G/EF^−^GCAP2 localized to rod outer segments (Fig. 10 histogram and Fig. S4, n = 13 cells). We believe that the reason that a 100% reversion was not observed is that 14-3-3 shows some affinity for unphosphorylated bEF^−^GCAP2 as well (Fig. 5). We therefore infer that 14-3-3 binding to phosphorylated GCAP2 retains the protein at proximal compartments, in what clearly represents an important step in the regulation of GCAP2 subcellular distribution *in vivo*, somewhat analogous to 14-3-3 regulation of phosducin availability during dark and light adaptation.

14-3-3 proteins are a family of phosphobinding proteins of about 30 kDa that comprises seven homologs in mammals. They exist as homo- or hetero-dimers that are rigid in structure, with each 14-3-3 dimer binding to two different phospho-binding sites either in the same or in two independent target proteins. By masking an epitope, clasping epitopes or promoting the scaffolding of their clients, 14-3-3 proteins exert a diverse range of regulatory roles in metabolism, trafficking or integration of cell survival versus cell death pathways [39]. In the retina, 14-3-3 proteins interact with phosducin at rod inner segments, regulating the amount of free phosducin during dark- and light-adaptation [44], [45]. Phosducin modulates the amount of Tr_αβγ_ heterotrimer through competition with Gt_α_ subunit for binding to the Gt_βγ_ complex. When light exposure activates Gt, releasing Gt_βγ_ from Gt_α_ at rod outer segments, phosducin association to Gt_βγ_ facilitates Gt_α_ and Pd-Gt_βγ_ independent translocation to the inner segment compartment [46]. At the inner segment during dark-adaptation phosducin is simultaneously phosphorylated at Ser-54 and Ser-73 residues by PKA and CaMK, which causes a competing interaction with the 14-3-3 protein that dramatically reduces phosducin binding to Gt_βγ_ [47]. This allows the redistribution of Gt_α_ and Gt_βγ_ to rod outer segments, the former assisted by UNC119 and the latter by PrBP [48], [49]. At rod outer segments Tr subunits are discharged to membranes and a heterotrimer forms again.

**Figure 11:**
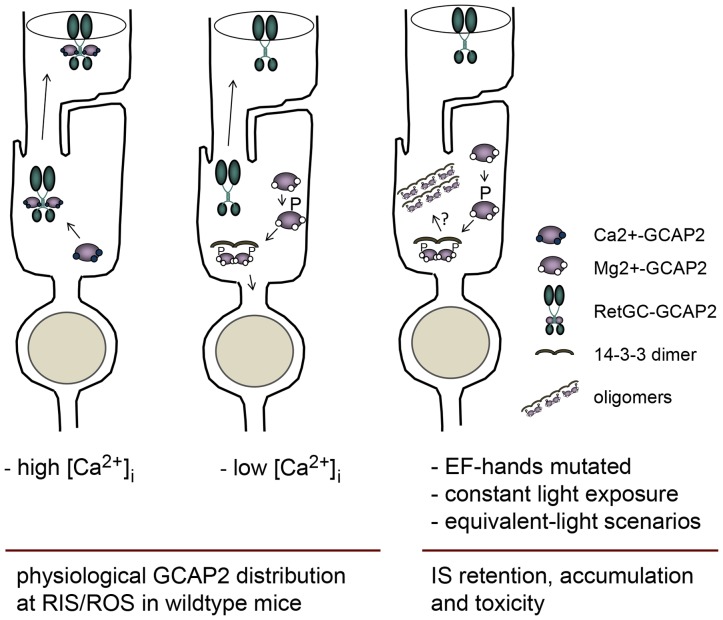
Model depicting a mechanism regulating GCAP2 distribution in rods involving GCAP2 phosphorylation and 14-3-3 binding. Under high Ca^2+^ conditions typical of the dark-steady state, the Ca^2+^-loaded form of GCAP2 would bind to Ret-GC and be transported to rod outer segments (left sketch); whereas under low Ca^2+^ conditions (e.g. light periods), the Ca^2+^-free form of GCAP2 would be retained by 14-3-3 at proximal compartments (middle). These alternating scenarios would result in approximately half of GCAP2 distributing to the inner segment and proximal compartments of the cell, and half to the outer segment compartment in a physiological situation (e.g. in wildtype mice raised in standard cyclic light). However, a prolonged light exposure or genetic conditions that would result in an abnormal accumulation of GCAP2 in its Ca^2+^-free form (e.g. mutations in components of the phototransduction cascade causing unabated signaling, hyperpolarization of the cell and a sustained reduction in [Ca^2+^]_i_ at the inner segment) would lead to neurodegeneration likely by inducing GCAP2 aggregation, or by GCAP2-mediated toxicity some other way (right sketch).

How 14-3-3 binding to phosphorylated GCAP2 fits with GCAP2 overall role in photoreceptor cell physiology and inherited retinal dystrophies is only emerging. It is clear from this work that GCAP2 is phosphorylated preferentially in its Ca^2+^-free form *in vivo*. Because it is well established that GCAP2 in its Ca^2+^-free form forms dimers [29], and that 14-3-3 exists as dimers that bind to two consensus binding sites in client proteins [39], it seems straightforward to propose that a dimer of 14-3-3 would bind to a dimer of GCAP2, presumably to stabilize it (Fig. 11). Because GCAP1 and GCAP2, unlike recoverin or phosducin, were shown not to redistribute between subcellular compartments during dark- or light-adaptation [50], we deduce that this mechanism would mainly affect the cytosolic distribution of newly synthesized protein.

We propose a model in which the GCAP2 molecules synthesized during the dark period (predominantly in the Ca^2+^-loaded state) would bind to RetGC and be transported to rod outer segments, whereas the GCAP2 molecules synthesized in the light period (GCAP2 in its Ca^2+^-free state) would be phosphorylated and retained at proximal compartments by 14-3-3 binding (Fig. 11). Such a scenario would result in the phosphorylation and retention to proximal compartments of about 50% of GCAP2 molecules in a physiological situation (wildtype mice raised in standard cyclic light). This is what we observe by IEF (Fig. 7) and by immunolocalization analysis (Fig. 4). This model would also explain why 12 h of dark-adaptation did not have a noticeable effect on the steady-state phosphorylation levels of GCAP2 (Fig. 7). The model would predict that wildtype mice reared under constant light exposure for a period of time covering the complete renewal of the rod outer segment would result in massive GCAP2 retention at the inner segment, given that GCAP2 would always be synthesized in a context of low [Ca^2+^]_i_, and that is precisely what we have observed after exposing mice to constant light for 30 days (Fig. 10). In contrast, it would be predicted that wildtype mice reared in constant darkness would result in GCAP2 major localization to the rod outer segment layer. This, however, was not observed. In mice reared in darkness for 30 days GCAP2 distributed about equally between inner and outer segment layers (Fig. 10). We interpret this result as an indication that some of the protein interactions required for GCAP2 transport to the rod outer segment (e.g. RetGC, RD3) might be rate-limiting.

We propose that GCAP2 phosphorylation and 14-3-3 binding constitute a major molecular determinant of GCAP2 subcellular localization upon its synthesis. What is its physiological relevance? A possibility is that 14-3-3 binding to GCAP2, by trapping GCAP2 to proximal compartments, might work to secure a reservoir of GCAP2 at these compartments, where GCAP2 may be exerting other roles, e.g. at the synaptic terminal [34], [35]. Alternatively, the 14-3-3 trapping of Ca^2+^-free GCAP2 upon its phosphorylation might serve as a protein quality control mechanism, to avoid that an excess of Ca^2+^-free, aggregation-prone GCAP2 molecules would reach the rod outer segment. Irrespective of its physiological significance, this regulatory enzymatic step is specific of GCAP2, given that GCAP1 is not phosphorylated, and might have evolved because it is more relevant for rods than cones. Conditions that substantially alter this regulatory mechanism increasing the protein retention at the inner segment of the Ca^2+^-free form would have toxic consequences for the cell. We propose that toxicity in this scenario would arise from GCAP2 natural tendency to aggregate (see below).

### Physiological implications of GCAP2 phosphorylation and 14-3-3 binding for inherited retinal dystrophies

GCAP2 phosphorylation and 14-3-3 binding are observed to a more moderate extent in the bGCAP2 control transgenic line (IEF gel in Fig. 7) than in wildtype mice, presumably because bovine GCAP2 is not such a good substrate for the murine kinase as the endogenous murine GCAP2. In wildtype mice phosphorylated GCAP2 at steady state constitutes about 50% of the total protein, consistent with about 50% of the protein retention at rod proximal compartments. This indicates that GCAP2 phosphorylation and 14-3-3 binding are not in itself toxic for the cell. It is therefore the deregulation of this mechanism– when all GCAP2 molecules are impaired to coordinate Ca^2+^ and GCAP2 phosphorylation and 14-3-3 binding are happening to a much larger extent- that correlates with severe retinal degeneration in the bEF^−^GCAP2 line.

How does the accumulation of GCAP2-14-3-3 complexes at the rod inner segment lead to cell death? We hypothesize that accumulation of these complexes might result in pathology due to the formation of misfolded GCAP2 oligomers, in much a similar way to which synuclein, APP, Tau, Huntingtin or ataxin lead to neuronal cell death in Parkinson's (PD), Alzheimer (AD), Huntington's (HD) or spinocerebellar ataxia (SCA) diseases. GCAP2 shows a natural tendency to aggregate. Structural studies have shown that the Ca^2+^-free form of GCAP proteins, and particularly of GCAP2, are difficult to maintain in solution and are prone to aggregation [51]. When expressed in bacteria, recombinant GCAP2 accumulates in inclusion bodies, is only solubilized at high concentrations of guanidinium or urea, and is difficult to maintain in solution after refolding [1]. Dimers and high molecular weight aggregates can typically be distinguished by SDS-PAGE, more prominently for EF^−^GCAP2 than for the wildtype form of the protein (e.g. this study, Fig. 5B). On the other hand, previous studies have found a close association between 14-3-3 and progressive neurodegenerative diseases. 14-3-3 proteins have been shown to colocalize with AD neurofibrillary tangles that are composed primarily of hyperphosphorylated tau proteins [52], [53]. In PD, 14-3-3 is detectable in Lewy bodies which accumulate α-synuclein [54]; and 14-3-3 colocalization was also reported for mutant ataxin in SCA [55]. Furthermore, 14-3-3 zeta and epsilon binding to phosphorylated ataxin-1 at S776 was shown to aggravate neurodegeneration by stabilizing mutant ataxin, retarding its degradation and enhancing its aggregation in transfected cells and transgenic flies [55]. The requirement of 14-3-3 zeta for Htt86Q aggregate formation has also been established in cells [56].

We propose that the mutant form of GCAP2 locked in its Ca^2+^-free conformation results in toxicity *in vivo* by the progressive formation of soluble high molecular weight oligomers of GCAP2-14-3-3 that are toxic for the cell. Inclusion bodies were not detected in our immunofluorescence assays with the polyclonal or monoclonal anti-GCAP2 antibodies used, or the anti-14-3-3ε monoclonal antibody. It may happen that these antibodies do not recognize inclusion bodies, or that their absence would result from a relatively efficient clearance of the mutant protein and therefore slow formation of putative deposits. In this sense we have observed that inhibition of the proteasome results in an increase of EF^−^GCAP2 levels (López-del Hoyo and Méndez, unpublished observation).

This mechanism of toxicity caused by GCAP2 misfolding may contribute to the pathology of genetic mutations causing “equivalent-light damage” that result in a sustained reduction in the level of intracellular Ca^2+^: e.g. mutations in the visual cycle resulting in opsin basal constitutive activity (e.g. null mutations in RPE65 [42]). Furthermore, this mechanism of toxicity is likely to contribute to cell death and retinal degeneration in those cases of Lebers Congenital Amaurosis (LCA) in which two conditions converge: GCAPs accumulation at the inner segment and a sustained reduction in the level of intracellular Ca^2+^. Those conditions are met, for instance, in LCA1 caused by null mutations in RetGC-E (GUCY2D) or LCA12 caused by mutations in RD3, two severe and prevalent inherited retinal dystrophies.

In conclusion, we propose that GCAP2 may be a mediator of “equivalent-light” genetic damage, by its natural tendency to aggregate when in its Ca^2+^-free form, in a process regulated by phosphorylation and 14-3-3 binding. Future studies will be addressed at further characterizing the stoichiometry, solubility and turn-over of GCAP2-14-3-3 complexes, as well as their effects on the normal functions of the cell.

## Materials and Methods

### Transgenic expression of bEF^−^GCAP2 in mouse rods and determination of transgene expression levels by western blot

This study was conducted in accordance with the ARVO statement for the Use of Animals in Ophthalmic and Vision Research and in compliance with Acts 5/1995 and 214/1997 for the welfare of experimental animals of the Autonomous Community (Generalitat) of Catalonia, and approved by the Ethics Committee on Animal Experiments of the University of Barcelona and the University of Southern California. The GCAP2 expression vectors used to generate transgenic mice were obtained by assembling the 4.4 kb mouse opsin promoter with bovine wildtype GCAP2 cDNA or mutant bEF^−^GCAP2 [GCAP2 E80Q/E116Q/D158N, [38]] cDNA (0.7 kb), and a 0.6 kb fragment containing the mouse protamine 1 polyadenylation sequence, into pBluescript II SK (Stratagene, La Jolla, California). The resulting fusion gene, 5.7 kb in size, was excised from the plasmid, gel purified and microinjected at 1 µg/ml into the pronuclei of C57Bl6/J×DBA/2J F1 hybrid mouse embryos (The Jackson Laboratories, Bar Harbor, Maine). Injected embryos were implanted into pseudopregnant females, and progenie was screened for founders by PCR amplification of tail genomic DNA with primers:

Rh1.1: 5′GTGCCTGGAGTTGCGCTGTGGG3′ (forward) and p24: 5′TGGCCTCCTCGTTGTCCGGGACCTT3′ (reverse).

Founder mice were bred to C57Bl6 mice to maintain the transgene in a pigmented wildtype genetic background, or to GCAPs−/− to generate GCAPs−/− bEF^−^GCAP2 mice. To detect transgenic GCAP2 expression by immunoblot, retinas from mice of each genotype were obtained at either postnatal day 22 (p22) (for WT and mice from line B) or p40 (lines A and E), and were homogenized in 100 µl of homogenization buffer [80 mM TrisHCl, pH 8.0, 4 mM MgCl_2_, 0.5 mg/ml Pefabloc SC, 0.5 mg/ml Complete Mini protease inhibitors (Roche, Basel, Switzerland)]. After addition of SDS Laemmli sample buffer, samples were boiled for 5 min, and fractions corresponding to 1/40 of a retina were resolved by SDS-PAGE in a 12% tris-glycine gel and transferred to nitrocellulose membranes (Protran, Schleicher & Schuell, Keene, NH). Membranes were incubated with polyclonal antibodies to bovine GCAP2 [p24ΔN [1], a gift from A. Dizhoor, Pennsylvania College of Optometry, Elkins Park, Pennsylvania], [GC1 and GC2 [57], a gift from D. Garbers, HHMI and UT Southwestern Medical Center, Dallas] and PDE (αβγ2, Cytosignal, Irvine, CA). Immunopositive protein bands were detected with a peroxidase-conjugated goat anti-rabbit IgG with the ECL system (Amersham, UK).

For determination of the precise level of expression of the transgene (expressed as a function of the endogenous), retinal extracts from mice from bEF^−^GCAP2 line B and line A (2-fold serial dilutions of retinal extracts obtained as described above) were directly compared to retinal extracts from the bGCAP2 control line (2-fold dilutions). The expression level of bGCAP2 in this line was previously established as 2-fold the endogenous levels [11]. This same study established that the anti-GCAP2 antibody used recognized bGCAP2 with a 1.5-fold higher affinity than mGCAP2 [11]. That is the reason that we compared transgenic bEF^−^GCAP2 to the transgenic bGCAP2 reference. The 2-fold serial dilutions in each sample were used to obtain the integration values of those bands present in the linear range in the same gel, for a direct comparison. The expression of bEF^−^GCAP2 line A was determined to be 2.76±0.12 –fold the endogenous levels (average ± St Dev, n = 3). The expression of bEF^−^GCAP2 line B was estimated to be 1.4-fold higher than line A, that is, 3.85-fold the endogenous levels.

### Histology and retinal morphometry

For histological analysis of the retina by light microscopy, eyecups were marked for orientation, fixed, embedded in epoxy resin and sectioned at 1 µm thickness as described previously [58]. Retinal morphometry measurements were taken as previously described [58].

### Guanylate cyclase assays

Guanylate cyclase activity was assayed in mouse retinal homogenates. Six retinas from dark-adapted mice of each genotype were dissected under infrared illumination, pooled and homogenized in 112 µl of 2× assay buffer (100 mM MOPS-KOH pH 7.5, 16 mM NaCl, 200 mM KCl, 2 mM IBMX, 20 mM MgCl_2_, 14 mM 2-β-mercaptoethanol). From this, 12.5 µl aliquots were mixed with either 7.5 µl of 1.33 mM EGTA (for a final concentration of 0.4 mM EGTA per reaction, the “low Ca^2+^” condition) or 7.5 µl of 6.6 µM CaCl_2_ (for a final concentration of 2 µM Ca^2+^, the “high Ca^2+^” condition) and preincubated at 30°C for 10 min. Reactions were initiated by addition of 5 µl of 5× substrate mix (1.0 mM GTP, 0.2 µCi/µl of [α-^32^P]GTP, 1.0 mM ATP), and allowed to proceed for 15 min at 30°C. Reactions were terminated by addition of 500 µl of ice-cold 120 mM Zn(OAc)_2_, neutralized with 500 µl of Na_2_CO_3_, kept at −80°C for 15 min and centrifuged at 14,000 g, 4°C for 20 min. Radiolabeled cGMP in the supernatants was separated from radiolabeled GTP by alumina column chromatography as described [59]. Protein concentration in retinal homogenates was determined by Bradford. Results are the average and standard deviation of four independent experiments performed in duplicate, with mice that were between p20 and p30. Guanylate cyclase activity was also determined in all retinal homogenates after the addition of 3 µM recombinant GCAP2 as a control for the presence of active Ret-GCs.

### Immunocytochemistry

To obtain retinal sections for immunofluorescence analysis mouse eyecups were fixed, infiltrated in sucrose or acrylamide, embedded in OCT and cryosectioned as described [34]. Sections were incubated with blocking solution (3% normal goat serum, 1% BSA, 0.3% Triton X100 in PBS pH 7.4, 1 h at room temperature); primary antibody (14 h at 4°C), secondary antibody (1 h at room temperature), and fixed for 15 min in 4% paraformaldehyde prior to being mounted with Mowiol [Calbiochem, Billerica, MA]. An antigen retrieval treatment of retinal sections [incubation in 0.05 mg/ml proteinase K in PBS pH 7.4 for 2 min at room temperature followed by a heat shock at 70°C for 10 sec] was needed for GCAP2 immunostaining. Antibodies used were: a polyclonal anti-GCAP2 (35), monoclonal anti-GCAP2 [mAb2235, Millipore, Billerica, MA], rabbit monoclonal anti-14-3-3ε [EPR3918, abcam, Cambridge, UK]. Secondary antibodies for immunofluorescence were Alexa 488 goat anti-rabbit IgG and Alexa 555 goat anti-mouse IgG [Molecular Probes, Eugene, Oregon]. Images were acquired at a laser scanning confocal microscope (Leica TCS-SL and TCS-SP2).

### GCAP2 immunoprecipitation and protein identification by LC-MS/MS

For GCAP2 immunoprecipitation in order to identify protein interacting partners in the different phenotypes [GCAPs−/− bGCAP2, GCAPs−/− bEF^−^GCAP2 and GCAPs−/− control mice] forty retinas per phenotype were pooled and homogenized in HEPES buffer [10 mM HEPES pH 8.0, 5 mM KCl, 135 mM NaCl, 1.5 mM MgCl_2_, 4 mM EGTA, 1 mM PMSF, 1 mM NaF, 1 mM β-mercaptoethanol, 1% Triton-X100 and protease inhibitor cocktail Complete Mini (Roche, Basel, Switzerland)], and clarified by centrifugation. Supernatants were incubated with anti-GCAP2 monoclonal antibody-covalently crosslinked to magnetic beads (Dynabeads, Life Technologies, Carlsbad, California) for 45 minutes at room temperature (anti-GCAP2 mAb2235, Millipore, Billerica, MA). Following extensive washing, elution was performed with 0.2M Glycine-HCl pH 2.5. Elution fractions were neutralized and concentrated by ethanol precipitation, reduced and alkylated with 45 mM DTT at 60°C followed by 100 mM iodoacetamide at room temperature, dehydrated and rehydrated with sequencing grade trypsin in 25 mM ammonium bicarbonate for 12 h. For LC-MS/MS samples were resuspended in 0.1% formic acid and injected into a series Proxeon LC nanoEASY system (Thermo Fisher Scientific, West Palm Beach, Florida) coupled to a LTQ-Velos Orbitrap (Thermo Fisher Scientific, West Palm Beach, Florida). The resulting mass spectral peak lists were searched with the Sequest search engine (v.2.1.04, Matrix Sciences, London, UK) against the merged BOVIN-MOUSE UP SP r 2011-1.fasta sequence library. Immunoprecipitation assays and LC-MS/MS analysis with the indicated mouse phenotypes were performed in three independent experiments, with similar results.

### *In vitro* phosphorylation of GCAP2 and pull-down assays with mock- or phosphorylated-GCAP2

For *in vitro* phosphorylation of GCAP2 in the presence of radioactivity, 20 µl reaction mixtures contained 8.5 µg of purified recombinant wildtype bGCAP2 or bEF^−^GCAP2, purified PKGIα (100 units, Calbiochem, Billerica, MA) and 3 µCi of ^33^P-γATP (Perkin Elmer, Massachusetts, USA) in phosphorylation reaction buffer (30 mM Tris-HCl pH 7.5, 5 mM MgCl_2_, 5 mM sodium phosphate buffer pH 7.5, 6 mM DTT, 0.1 mM EGTA and 10 µM ATP). For reactions in Ca^2+^ or EGTA conditions, the 0.1 mM EGTA in the reaction buffer was substituted to 5 mM CaCl_2_ or 2 mM EGTA, respectively. cGMP was added to 500 µM (to obtain phosphorylated GCAP2 or P-GCAP2) or not added (mock- controls). After incubation for 2 h at 30°C and overnight at 4°C, each reaction mixture was diluted with Laemmli buffer and resolved by 15% SDS-PAGE. Following transfer to a nitrocellulose membrane, an autoradiograph of the ^33^P phosphorylation products was obtained by 15 min of exposure to a Kodak X-ray film. The nitrocellulose membrane was subsequently incubated with a pAb anti-GCAP2 and IRDye 800CW Goat Anti-rabbit IgG for GCAP2 immunodetection.

To obtain phosphorylated bGCAP2 or bEF^−^GCAP2 for pull-down assays in the absence of radioactivity, the same procedure was used except that 25 µg of bGCAP2 or bEF^−^GCAP2 protein and 230 units of purified PKGIα were used per reaction tube. The product of each reaction tube was cross-linked to 2.5 mg of magnetic beads (Life Technologies, Carlsbad, California) and used in pull-down assays with solubilized bovine retina. Each sample was incubated with material corresponding to 1/8 of a bovine retina, previously homogenized in binding buffer (10 mM HEPES, 135 mM NaCl, 5 mM KCl, 1 mM PMSF, 1 mM NaF, 1 mM β-mercaptoethanol, 1% Triton X-100, 4 mM EGTA, 2 mM EDTA, Complete Mini protease inhibitors, pH 7.4) and pre-cleared by centrifugation. After 1 h incubation at room temperature, beads were washed and bound proteins were eluted under acidic conditions, equilibrated and ethanol precipitated. Samples were resolved by 15% SDS-PAGE and transferred to a nitrocellulose membrane. For Western detection of GCAP2 and 14-3-3 the following antibodies were used: a polyclonal anti-GCAP2 (35), a pAb to 14-3-3pan (JP18649, IBL International, Hamburg, Germany), a mAb to 14-3-3ε (EPR3918, abcam, Cambridge, UK), a IRDye 800CW Goat anti-rabbit IgG and a IRDye 680CW Goat anti-mouse IgG (Tebu Bio, Offenbach, Germany). Image was acquired at the Odyssey Imaging System (LI-COR, Lincol, Nebraska USA).

### *In situ* phosphorylation assays

All mice for *in situ* phosphorylation assays were 30–36 days old. Mice were dark-adapted for a minimum of 14 h prior to use. Retinas were dissected under infrared light (two retinas per phenotype per light condition) and incubated for 90 min in 600 µl of bicarbonate-buffered Locke's solution (112.5 mM NaCl, 3.6 mM KCl, 2.4 mM MgCl_2_, 1.2 mM CaCl_2_, 10 mM HEPES, 0.02 mM EDTA, 20 mM NaHCO_3_, 10 mM glucose, 3 mM sodium succinate, 0.5 mM sodium glutamate, 0.1% vitamin and amino acids supplement) containing 1 mCi/ml [^32^P_i_]H_3_PO_4_ (10 mCi/ml, Perkin Elmer, Massachusetts, USA) in the dark in a 5% CO_2_ incubator to allow incorporation of ^32^P in the endogenous ATP pool. Following incubation, retinas were washed with Locke's solution and immediately homogenized in 200 µl of solubilization buffer [10 mM HEPES, 135 mM NaCl, 5 mM KCl, 1 mM PMSF, 2 mM NaF, 4 mM EGTA, 1.5 mM MgCl_2_, 2 mM EDTA, 1% Triton X100, complete mini protease inhibitors (Roche Applied Sciences, Basel, Switzerland), pH 7.4] in the dark, or exposed to bright white light for 5 min prior to homogenization. Samples were clarified by centrifugation at 13,000 g for 20 min at 4°C and supernatants were transferred to new tubes. From these samples, 10 µl aliquots were resolved by 15% SDS-PAGE to obtain an autoradiograph of the input samples. Visualization of inputs required 4 h of exposure with a Kodak X-ray film. The remaining volume of samples (180 µl) were used to immunoprecipitate GCAP2 with an anti-GCAP2 monoclonal antibody (anti-GCAP2 mAb2235, Millipore, Billerica, MA) coupled to magnetic beads (Dynabeads, Life Technologies, Carlsbad, California) as described above. After acidic elution of bound fractions, samples were neutralized and proteins precipitated with ethanol. Protein pellets were resolved by 15% SDS-PAGE and transferred to a nitrocellulose membrane. Visualization of phosphorylated proteins in the bound fractions by autoradiography required 4 days of exposure with a Kodak X-ray film. The membrane was subsequently incubated with a polyclonal antibody anti-GCAP2 and IRDye 800CW Goat Anti-rabbit IgG; and a polyclonal antibody to 14-3-3pan (JP18649 IBL International, Hamburg, Germany) and IRDye 680CW Goat Anti-mouse IgG, and scanned at an Odyssey Image Acquisition system (LI-COR, Lincoln, Nebraska USA).

### Analysis of steady-state GCAP2 phosphorylation by isoelectrofocusing (IEF) gels

Retinas from mice of the indicated phenotypes were dissected under infrared light, and each retina was solubilized in 150 µl of buffer (10 mM Hepes pH 7.5, 1 mM MgCl_2_, 10 mM NaCl, 0.1 mM EDTA, 1% dodecyl-maltoside, 1 mM DTT, 50 mM NaF) overnight at 4°C. Samples were centrifuged at 14000 rpm for 5 min, 15 µl of the supernatant was loaded onto an isoelectrofocusing gel (pH range 3–8) on a Pharmacia FBE 300 flat bed apparatus, and focused for 2 h at 23W. Proteins were transferred to a nitrocellulose membrane by capillary action and incubated with GCAP2 pAb. Bands were visualized with the ECL system (Pharmacia).

### Transient transgenic expression of bS201G/EF^−^GCAP2 mutant in the retina by *in vivo* electroporation of plasmid DNA following its injection in the subretinal space

The expression vector for the mutant bS201G/EF^−^GCAP2 was obtained by site-directed mutagenesis of the expression vector described above for bEF^−^GCAP2 based on the 4.4 kb version of the mouse opsin promoter. Site-directed mutagenesis was performed with the QuikChange II site-directed mutagenesis kit (Agilent, Santa Clara, CA, USA) using primers:

bGCAP2_S201G_Fw: CTCAGCAGAGGCGGAAAGGTGCCATGTTC; bGCAP2_S201G_Rv: GAACATGGCACCTTTCCGCCTCTGCTGAG;

Mutagenesis was confirmed by sequencing. Mice were electroporated at p0 according to reference [40] and processed at p28–30. Briefly, 0.5 µl at a concentration of 6 µg/µl of DNA mix in PBS was injected into the subretinal space, by making use of a Nanojet microinjector and micromanipulator (Drummond Scientific, Broomall, PA). The DNA mix consisted of the expression vector for the specific GCAP2 mutant (bGCAP2, bEF^−^GCAP2 or bS201G/EF^−^GCAP2) in circular form and a tracer plasmid (pL_UG that expresses the green fluorescent protein (GFP) driven by the Ubiquitin C promoter, [60]) also in circular form, at a mass ratio of 2∶1. Electroporation was performed with a square-wave electroporator (CUI21, Nepagene, Japan) by triggering 5 pulses of 80 V with a 50 ms duration and an interval time of 950 ms. Electroporated pups were raised under standard cyclic light conditions and sacrificed at p28–30 for immunofluorescence analysis. Briefly, eyes were fixed in 4% paraformaldehyde in PBS at pH 7.4, embedded in acrylamide mix and frozen as described [34]. Retinal cryosections were obtained at 22 µm thickness. An antigen retrieval protocol was performed preceding the immunofluorescence studies: glass slides were incubated with proteinase K in PBS pH 7.4 (0.05 mg/ml) for 2 min and heated at 70°C for 8 sec. Sections were incubated in blocking solution (1% BSA, 3% normal goat serum, 0.1% Triton X100, PBS pH 7.4); primary antibody solution (1% BSA, 3% normal goat serum, PBS pH 7.4 containing 0.01 mg/ml polyclonal antibody to GCAP2 and 0.00025 mg/ml mAb 1D4 to rhodopsin); and secondary antibody solution (1% BSA, 3% normal goat serum, PBS pH 7.4 containing Alexa Fluor 647 anti-rabbit IgG (signal converted to red in figures); and Alexa Fluor 555 anti-mouse IgG (signal converted to blue in figures). Images were acquired in a Leica confocal microscope. GCAP2 and rhodopsin signal profiles were obtained for the individual cells shown by tracing a line along the inner segment compartment, and another line along the outer segment compartment, and plotting the summation of the red and the green signal along both lines from the collection of planes in a z-stack that covers the whole volume of the cell, by using the Leica confocal software (Leica Microsystems).

### Analysis of the effect of 30 days of constant light exposure versus 30 days of dark rearing on GCAP2 subcellular distribution in wildtype mice

Three-month old C57Bl/6J mice were kept in a ventilated dark cabinet for 30 d, or kept under constant light exposure for the same time (fluorescent light, 700 lux intensity inside the cage), and their eyecups were processed for immunofluorescence analysis. Retinal cryosections were stained with an anti-GCAP2 pAb and anti-rhodopsin mAb 1D4, by indirect immunofluorescence staining with Alexa 488 goat anti-rabbit IgG and Alexa 555 goat anti-mouse IgG (Molecular Probes, Eugene, Oregon). Z-axis stacks were obtained from four mice per condition at a Leica TCS-SL confocal microscope, and the percentage of GCAP2 signal colocalizing with the rhodopsin signal at the rod outer segment was quantified by ImageJ in three representative planes from each stack, one stack per mouse, four mice per condition. Precisely, for a 63×-objective frame of retina, an ROI was defined to include the inner and outer segment layers, while another ROI was delimited to the outer segment layer based on rhodopsin staining. GCAP2 signal was quantified in each ROI, and the percentage of GCAP2 colocalizing with rhodopsin was expressed as a function of GCAP2 at the inner and outer segment layers (ImageJ). Mean values were obtained and statistical analysis performed with GraphPad Prism 6.

### Electroretinogram

Electroretinogram responses to flash stimuli were recorded on a Nicolet Electrovisual Diagnostic System. Mice were dark-adapted for 12 h and then anesthetized under dim red light by intraperitoneal administration of Ketamine HCl (100 mg/kg) and Xylazine HCl (10 mg/kg). Phenylephrine HCl (2.5%) and Tropicamide (0.5%) were applied to the cornea to dilate the pupils, and mice were dark-adapted again for 10 min previous to the recording. Following administration of Tetracaine HCl (0.5%) eyedrops as a topical corneal anesthetic, the mice are placed on a heated pad at 37°C in a Faraday cage. The corneal electrode consisted of a carbon-fiber moistened in saline. A 1 ms light flash was delivered through a fiber optic centered vertically over a few millimeter of the corneal surface. Mice from the different genotypes were recorded over the course of eight months under identical conditions.

### Effect of constant light-rearing versus constant dark-rearing on retinal morphology of GCAPs−/− bEF^−^GCAP2 and GCAPs−/− bGCAP2 control mice

#### Evaluation of the rate of retinal degeneration by retinal morphometry analysis

The indicated mice were either reared in constant darkness or under constant light exposure (fluorescent light, 1500 lux intensity) and processed for analysis at postnatal day 20 or 40. Eyecups were marked for orientation, embedded in epoxy resin and section at 1 µm thickness as described previously [34].

Measurements of ONL thickness were taken by making use of a camera lucida attached to a microscope, with the aid of a graphics tablet (WACOM, Vancouver, WA) and the Axiovision LE Rel.4.1. imaging software (Carl Zeiss Inc., Germany). A stage micrometer (Klarmann Rulings, Litchfield, NH) was used for calibration. Each retina half (superior and inferior) was divided into ten equal segments from the optic nerve to the tip (excluding a radius of 100 µm from the optic nerve, due to the natural thinning of the ONL at this region). Three independent measurements were taken per segment, and the average value was obtained. In this manner 60 independent measurements were obtained per section (20 segments×3 measurements/segment). The mice analyzed per genotype were: GCAPs−/− bEF^−^GCAP2 line A, n = 4 for each dark and light conditions; GCAPs−/− bGCAP2 line E, n = 3 for each dark and light conditions; and GCAPs −/− control line, n = 2 for each condition.

## Supporting Information

Figure S1 Determination of bEF^−^GCAP2 transgenic expression levels in lines A and B. **A.** The level of expression of bEF^−^GCAP2 in line A was determined by direct comparison with that of bGCAP2 in line E, by loading in the same gel two-fold serial dilutions of a retinal homogenate representing 1/40 of a retina. Expression of bEF^−^GCAP2 was determined to be 1.38-fold higher (±0.06 St Dev) than that of bGCAP2 in control line E. Because line E was previously established to express 2-fold the endogenous levels of GCAP2 [11], line A is determined to express 2,76-fold the endogenous levels of GCAP2. **B.** Likewise, by comparison to line A, line B was determined to express 1.4-fold more transgene, or 3.86-fold the endogenous GCAP2 levels.
(TIF)

Figure S2 Timecourse for the loss of visual function in bEF^−^GCAP2 expressing mice as assessed by electroretinogram. ERG B-wave amplitudes (µV) are plotted to postnatal age of mice (months). Representative ERG responses are shown for each phenotype at 4 and 7.5 months of age.
(TIF)

Figure S3 Light-rearing does not prevent or delay retinal degeneration in bEF^−^GCAP2 transgenic mice. Rationale. If bEF^−^GCAP2 expression led to unabated cGMP synthesis *in vivo* and accumulation of cGMP was the basis of the pathology, then conditions of constant light exposure would slow the retinal degeneration by causing the sustained activation of PDE6 activity and cGMP hydrolysis. Light induced sustained cGMP hydrolysis would counteract unabated cGMP synthesis. **A.** Statistical comparison of ONL thickness at fixed regions along the central retina between bEF^−^GCAP2 transgenic mice reared in complete darkness or under constant light exposure (1,500 lux fluorescent light) at postnatal day 40. Measurements of ONL thickness (µm) were taken at ten equal intervals along the superior and inferior hemispheres of the retina, indicated in abscissas as positive values (superior retina) and negative values (inferior retina) from the optic nerve (position 0). The superimposition of the red and black lines indicate that retinal degeneration (shortening of ONL thickness along the retina) was observed to the same extent in dark-reared or constant light-reared bEF^−^GCAP2 mice. ONL thickness in GCAPs−/− bGCAP2 line E control mice are shown as a reference of normal retina values. **B.** Representative pictures from central superior retinas of dark-reared and constant light-reared GCAPs−/− bEF^−^GCAP2 line A and control mice at 20 and 40 postnatal days.
(TIF)

Figure S4 Additional images and GCAP2 staining profiles of cells from electroporated mice. Photoreceptor cells from electroporated mice with bGCAP2 (**A**, 1 cell), bEF^−^GCAP2 (**B**, 6 cells), and bS201G/EF^−^GCAP2 (**C**, 9 cells). GCAP2 stained in green, rhodopsin in red.
(TIF)

Table S1 Spectral counting of Proteins identified by LC-MS/MS in GCAP2 immunoprecipitation experiments.
(XLSX)
