# Correction: Functional EF-Hands in Neuronal Calcium Sensor GCAP2 Determine Its Phosphorylation State and Subcellular Distribution *In Vivo*, and Are Essential for Photoreceptor Cell Integrity

**DOI:** 10.1371/journal.pgen.1004744

**Published:** 2014-10-17

**Authors:** The PLOS Genetics Staff

Figure 1 is incorrect. In panel D the bGCAP2E 3m panel (top right) is identical to the bEF-GCAP2A 40d panel (middle left). The authors have provided a corrected version here.

**Figure 1:**
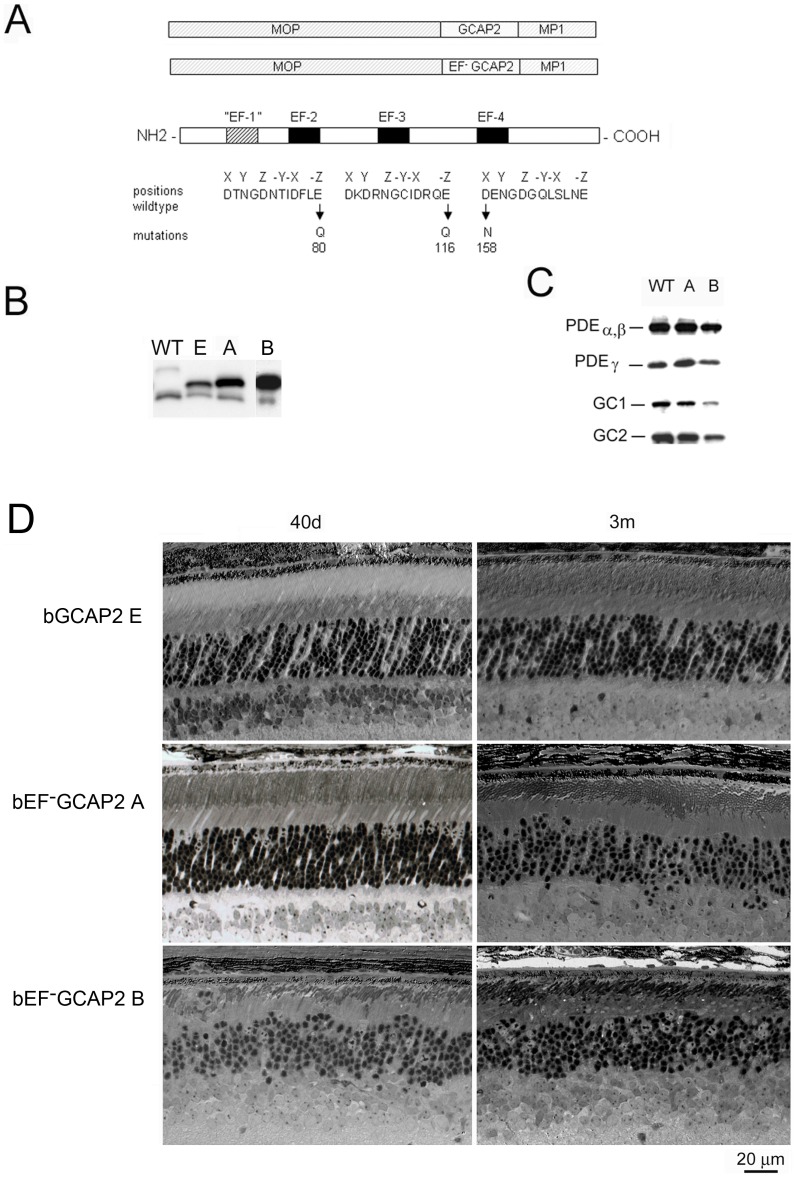
Transgene expression of bEF^−^GCAP2 in rods leads to retinal degeneration. A. Design of transgene expression vector. The cDNA of bovine GCAP2 with the three functional EF hands disrupted [GCAP2 (E80Q, E116Q, D158N) or bEF^−^GCAP2] was expressed under the mouse opsin promoter (MOP), with the polyadenilation signal of the mouse protamine 1 (MP1) gene. B. Western showing the level of expression of the transgene in bGCAP2 line E and bEF^−^GCAP2 lines A and B, compared to wildtype mice. Equivalent fractions of a retina were resolved by SDS-PAGE from wt (22 d of age), line E (40 d) and lines A (40 d) and B (22 d, showed from independent gel). An earlier time point was chosen for the strongest line (B) to reduce the effect that its rapid retinal degeneration has on total retinal protein content. Bovine and murine GCAP2 differ in size by three amino acids and can be distinguished by mobility. C. Compensatory changes in proteins involved in cGMP metabolism were not observed. The levels of PDEα,β and γ subunits, or GC1 and GC2 were mostly unaffected in mice from line A, whereas a reduction in all proteins was observed in line B at 22 d of age, due to the shortened outer segments in this line. D. Light micrographs of retinal sections from mice expressing bGCAP2 (line E) or bEF^−^GCAP2 transgene (lines A and B) in the GCAPs+/+ background at 40 d or 3 m.
