# Supplementary figures and images for: Functional EF-Hands in Neuronal Calcium Sensor GCAP2 Determine Its Phosphorylation State and Subcellular Distribution In Vivo, and Are Essential for Photoreceptor Cell Integrity
Source: PLoS Genet. 2014 Jul 24;10(7):e1004480. doi: 10.1371/journal.pgen.1004480 (PMC4109901; doi:10.1371/journal.pgen.1004480)

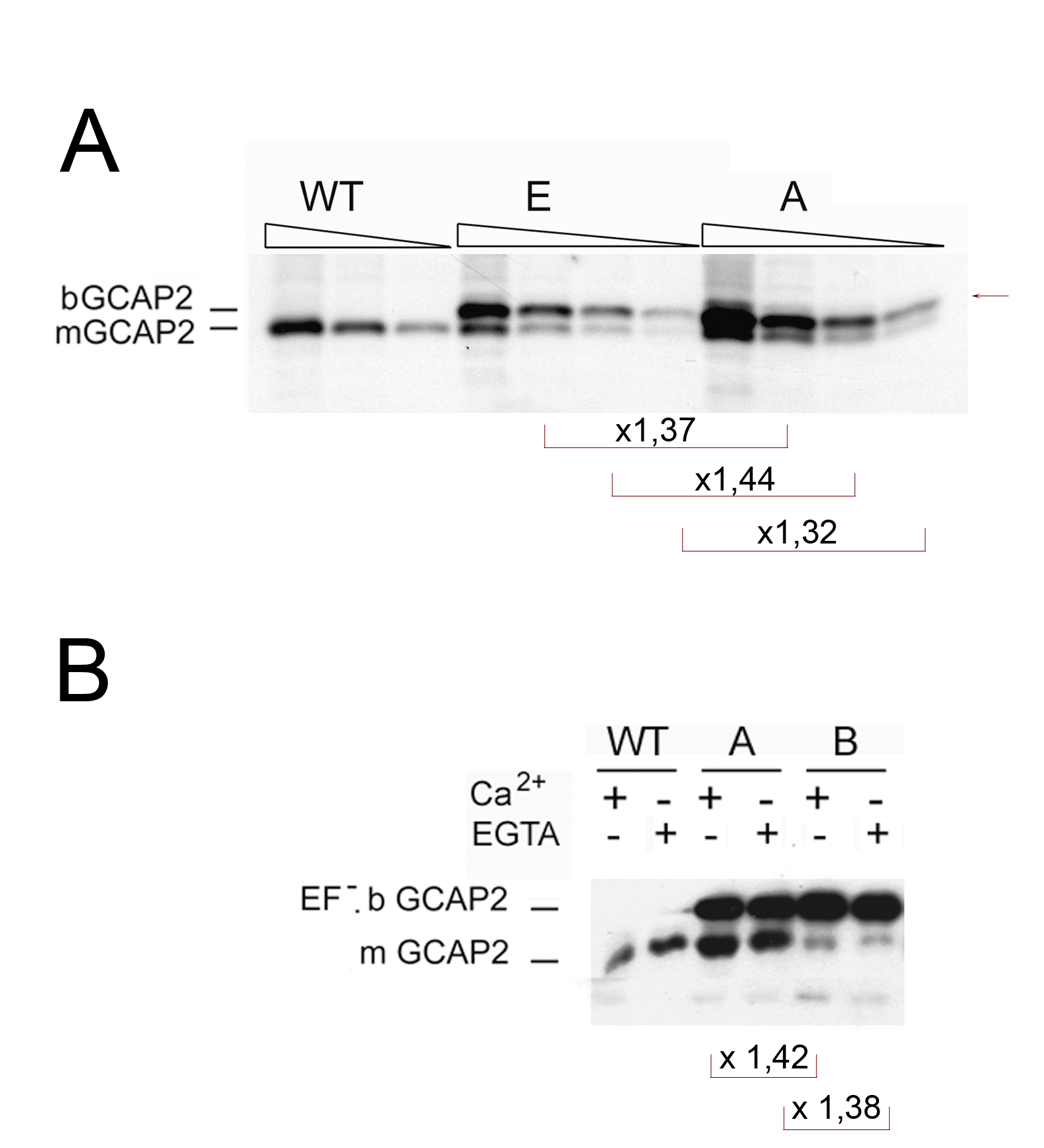

Supplement: Figure S1 — Determination of bEF−GCAP2 transgenic expression levels in lines A and B. A. The level of expression of bEF−GCAP2 in line A was determined by direct comparison with that of bGCAP2 in line E, by loading in the same gel two-fold serial dilutions of a retinal homogenate representing 1/40 of a retina. Expression of bEF−GCAP2 was determined to be 1.38-fold higher (±0.06 St Dev) than that of bGCAP2 in control line E. Because line E was previously established to express 2-fold the endogenous levels of GCAP2 [11], line A is determined to express 2,76-fold the endogenous levels of GCAP2. B. Likewise, by comparison to line A, line B was determined to express 1.4-fold more transgene, or 3.86-fold the endogenous GCAP2 levels. (TIF) [file pgen.1004480.s001.tif]

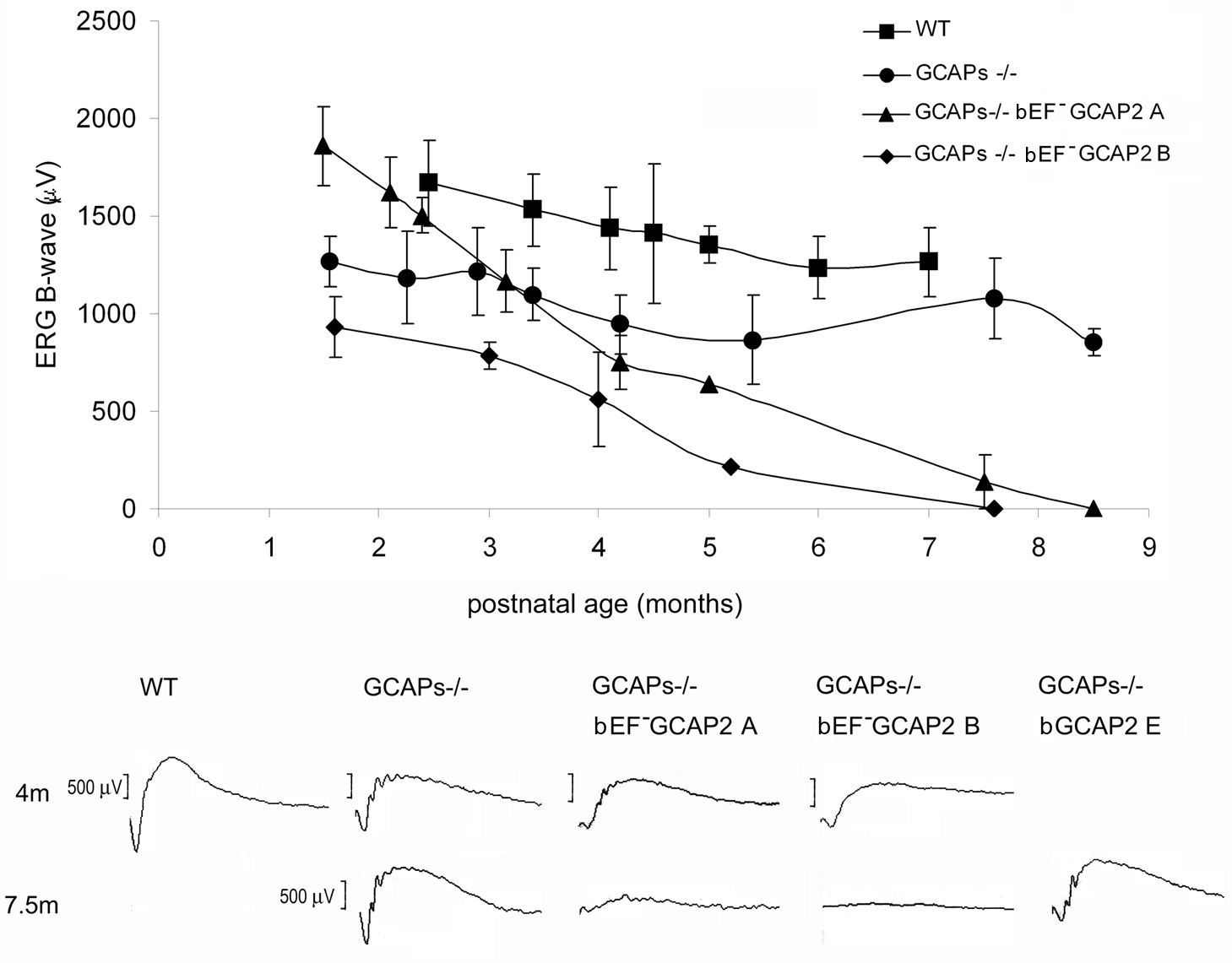

Supplement: Figure S2 — Timecourse for the loss of visual function in bEF−GCAP2 expressing mice as assessed by electroretinogram. ERG B-wave amplitudes (µV) are plotted to postnatal age of mice (months). Representative ERG responses are shown for each phenotype at 4 and 7.5 months of age. (TIF) [file pgen.1004480.s002.tif]

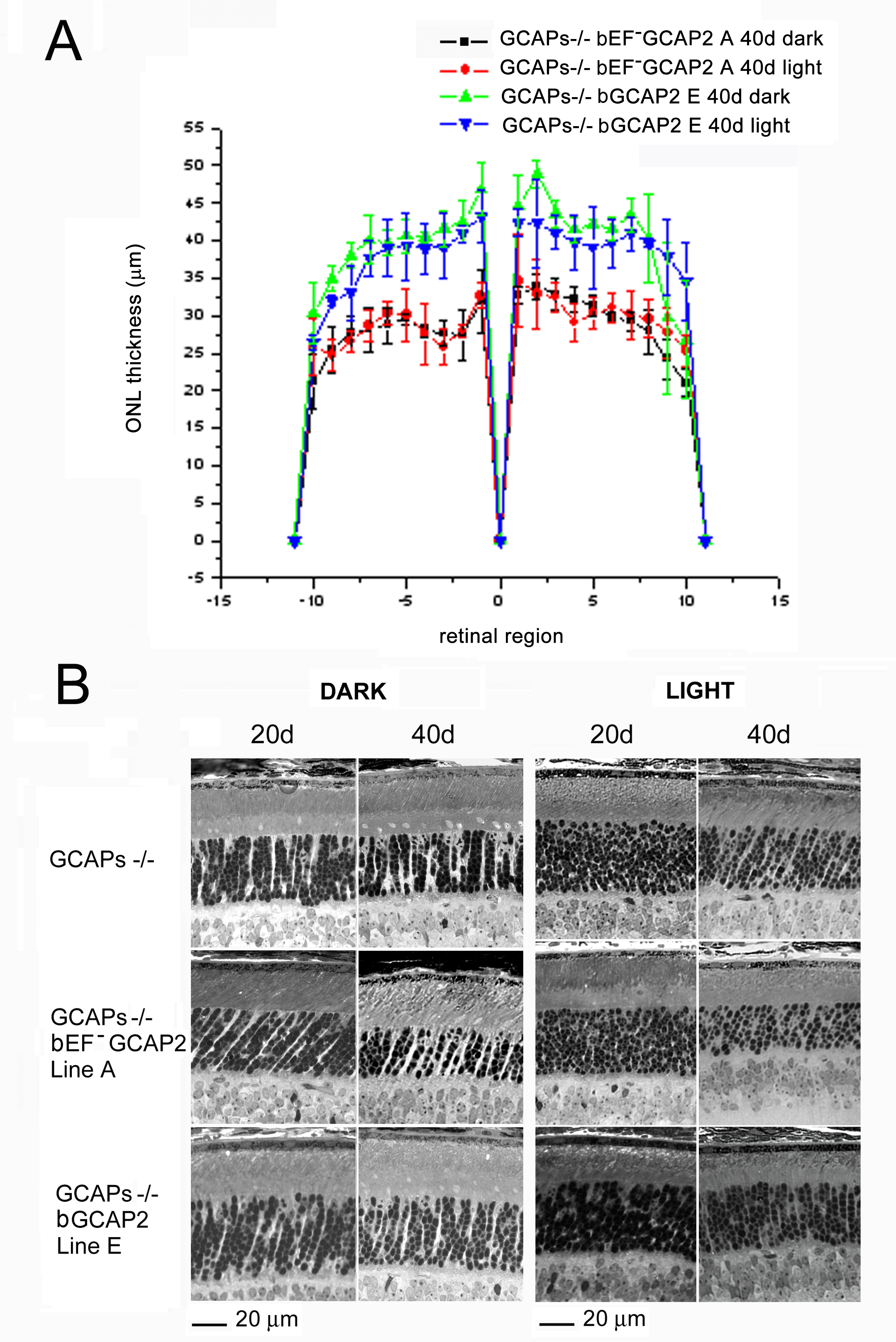

Supplement: Figure S3 — Light-rearing does not prevent or delay retinal degeneration in bEF−GCAP2 transgenic mice. Rationale. If bEF−GCAP2 expression led to unabated cGMP synthesis in vivo and accumulation of cGMP was the basis of the pathology, then conditions of constant light exposure would slow the retinal degeneration by causing the sustained activation of PDE6 activity and cGMP hydrolysis. Light induced sustained cGMP hydrolysis would counteract unabated cGMP synthesis. A. Statistical comparison of ONL thickness at fixed regions along the central retina between bEF−GCAP2 transgenic mice reared in complete darkness or under constant light exposure (1,500 lux fluorescent light) at postnatal day 40. Measurements of ONL thickness (µm) were taken at ten equal intervals along the superior and inferior hemispheres of the retina, indicated in abscissas as positive values (superior retina) and negative values (inferior retina) from the optic nerve (position 0). The superimposition of the red and black lines indicate that retinal degeneration (shortening of ONL thickness along the retina) was observed to the same extent in dark-reared or constant light-reared bEF−GCAP2 mice. ONL thickness in GCAPs−/− bGCAP2 line E control mice are shown as a reference of normal retina values. B. Representative pictures from central superior retinas of dark-reared and constant light-reared GCAPs−/− bEF−GCAP2 line A and control mice at 20 and 40 postnatal days. (TIF) [file pgen.1004480.s003.tif]

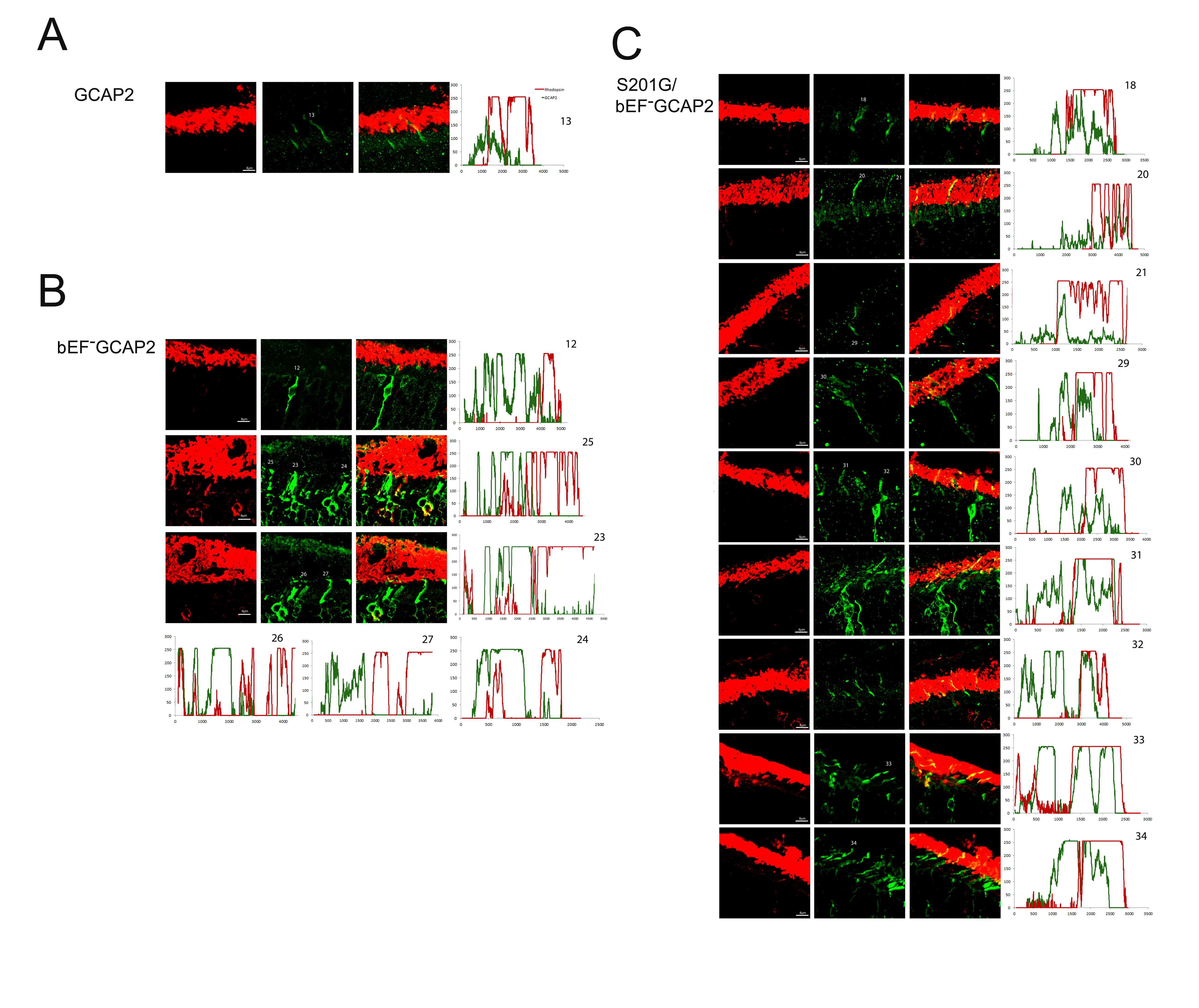

Supplement: Figure S4 — Additional images and GCAP2 staining profiles of cells from electroporated mice. Photoreceptor cells from electroporated mice with bGCAP2 (A, 1 cell), bEF−GCAP2 (B, 6 cells), and bS201G/EF−GCAP2 (C, 9 cells). GCAP2 stained in green, rhodopsin in red. (TIF) [file pgen.1004480.s004.tif]
